# Supplementary material for: Beyond six feet: The collective behavior of social distancing
Source: PLoS One. 2024 Sep 13;19(9):e0293489. doi: 10.1371/journal.pone.0293489 (PMC11398703; doi:10.1371/journal.pone.0293489)

Simulation Results 1: In Small-World Social Networks

Results in SR1-Table-1:

The table contains the equilibrium strategies for a set of distancing games computed based on the general theory on population games, which assumes that the population is large and well mixed, i.e., every individual can interact with all others in the population. Assume that there are n activities, and the activities are independent. Let $x = \{x_i : i = 1, .., n\}$ be the distancing strategy of an individual and $y = \{y_i : i = 1, …, n\}$ the average strategy of the population. Let $p_i(y) = w_i\sigma_i(y_i)$ be the potential distancing risk at activity i, where w_i is the risk factor of activity I and σ_I is a logistic function. Then, the equilibrium strategy of the game can be obtained with a formula, $x_i^* = \sigma_I^{-1}(\lambda/w_i)$ for all i where λ is a constant such that $1 = \Sigma_i \sigma_i^{-1}(\lambda/w_i)$ (details in Methods – Equilibrium strategies and stabilities).

The games are defined for 20 CASA activities. Therefore, n = 20, and $w_i = \delta_i \alpha_i + (1 - \delta_i) \beta_i$, where α_i and β_i are contact factor and impact factor for activity i, respectively, and δ_i is a severity parameter, $0 \leq \delta_i \leq 1$. The same logistic function σ_i is used for all i with $\kappa_i = 10$ and $\theta_I = 0.5$. The CASA activities and their estimated contact and impact factors are described and given in Table 1 in the main text. By setting δ_i to 0, 0.25, 0.5, 0.75, 1 for all i, five games are defined, and their equilibrium strategies are computed using the above formula. For each game (corresponding to a specific value for δ_I), the participating frequencies at equilibrium are listed in SR1-Table-1. The frequencies are represented by the active times in hours per week (112 active hours in total).

Results in SR1-Table-2:

This table shows the convergence of the distancing strategies to their equilibrium strategies in small world social networks. The network parameters are fixed for m = 2000, K = 6, and b = 0.3. The neighborhood size is varied from 1 to 2, 3, 4, 5, 6. Five games are simulated with δ_i set to 0, 0.25, 0.5, 0.75, 1 for all i, correspondingly. The strategies obtained from the simulation are compared with their equilibrium strategies. The average Euclidean norms of the differences between the individual strategies and their equilibrium strategies are listed.

The simulation is started with every individual assigned an initial strategy x randomly generated around the corresponding equilibrium strategy x*, i.e., for every individual, and for every activity i, x_i^* is first perturbed randomly by 20%; then an initial frequency x_i is generated randomly within 100% of deviation from the perturbed value of x_i^*. The simulation then proceeds and terminates, in most cases, in less than 20 generations, when either the average difference between the individual strategy and the equilibrium strategy is small enough or no strategy is improved in a few consecutive generations.

Each simulation is repeated for five times with five different sets of randomly generated initial strategies for all the individuals. The results in the table are the average outcomes. They show that the distancing strategies from the simulation consistently converge to their equilibrium

strategies for all the δ_i values and for all neighborhood sizes greater than 1. The converge is not as accurate when the neighborhood size is too small (k = 1).

Results in SR1-Table-3:

This table shows the convergence of distancing strategies to their equilibrium strategies in small world social networks. A single game is simulated with δ_i fixed to 0.5 for all i, the network parameters m fixed to 2000 and K to 6, but the randomness parameter b is changed from 0.1 to 0.2, 0.3, 0.4, 0.5. When b is smaller, the neighborhood of each network node tends to be more local or in other words, the connections among the individuals are shorter-ranged and less random. The game is also simulated with different neighborhood sizes, k = 1, 2, 3, 4, 5, 6. The strategies obtained from the simulation are compared with their equilibrium strategies. The average Euclidean norms of the differences between the individual strategies and their equilibrium strategies are listed.

The simulation is again started with every individual assigned an initial strategy x randomly generated around its equilibrium strategy x*, i.e., for every individual, and for every activity i, x_i* is first perturbed randomly by 20%; x_i is then generated randomly within 100% of deviation from the perturbed value of x_i*. Each simulation is repeated for five times. The results in the table are the average outcomes.

The simulation results do not show significant differences with different levels of randomness of the social network.

Results in SR1-Table-4:

This table shows the convergence of the distancing strategies to their equilibrium strategies in small world social networks. A single game is simulated with δ_i fixed to 0.5 for all i and the network parameters m fixed to 2000, K to 6, and b to 0.3. However, the way to generate the initial strategies is varied: For every individual, and for every activity i, x_i* is perturbed randomly by ρ = 0%, 10%, 20%, 30%, 40%, or 50%; and the initial frequency x_i is generated randomly within 100% of deviation from the perturbed value of x_i*.

The game is then simulated with the neighborhood size varied from 1 to 2, 3, 4, 5, 6. The strategies obtained from the simulation are compared with their equilibrium strategies. The average Euclidean norms of the differences between the individual strategies and their equilibrium strategies are listed. Each simulation is repeated for five times. The results in the table are the average outcomes. The results show that the simulation would converge as expected if the distribution of the initial strategies is not too far from the equilibrium strategy (ρ < 50%).

Results in SR1-Figure-1:

This figure contains the snapshots from the simulation for the distancing game in a small world social network. The parameters for the game are fixed to $\delta_i = 0.5$ for all i and $\rho = 20\%$, and for the network to m = 2000, K = 6, and b = 0.3. There are two columns of plots in the figure. The first one is from the simulation with the neighborhood size k = 1. The second one is from the simulation with the neighborhood size k = 2.

In each column, the first four plots show the changes of the individual strategies in four different generations. In each of the plots, along the x-axis are the 20 CASA activities. Over each activity, there are 2000 circles corresponding to the participating frequencies in this activity by the 2000 individuals. Along the y-axis are the participating frequencies for the activities represented by the active times in hours per week (112 active hours in total).

The last plot in each column shows the changes of the average difference between the individual strategy and the equilibrium strategy. As shown in the first column, for k = 1, the individual strategies are not improved after first few generations. The average difference between every individual strategy and the equilibrium strategy cannot be reduced further after 21 generations when the simulation is terminated.

However, when k = 2, as shown in the second column, the individual strategies converge quickly in several generations. The first plot shows the initial strategies at the 1st generation when they appear to be quite random; the second plot shows the strategies in the 4rd generation when they start converging; the third plot shows the strategies in the 7th generation when they almost converge to their equilibrium positions; the fourth plot shows the strategies in the 10th generation when they are close enough to their equilibrium values, and the simulation is terminated. As shown in the last plot, the average difference between every individual strategy and the equilibrium strategy is decreased as the generation increases and is eventually reduced to $< 10^{-4}$.

Results in SR1-Figure-2 and SR1-Figure-3 are produced in the same way as those in SR1-Figure-1 except that SR1-Figure-2 shows the results for k = 3, 4 and SR1-Figure-3 for k = 5, 6. The results for k = 3, 4, 5, 6 are all similar with those for k = 2, showing that the simulated distancing strategies converge to the equilibrium strategies for all neighborhood sizes greater than or equal to 2.

SR1-Table-1. Equilibrium strategies x* / y* for distancing games with 20 CASA activities

Act \ δ_i =	0.00	0.25	0.50	0.75	1.00
1	5	7.1175	9.1731	11.3984	14
2	5	7.1175	9.1731	11.3984	14
3	3	5.3571	7.7534	10.5003	14
4	3	5.3571	7.7534	10.5003	14
5	5	5.9203	6.5075	6.8719	7
6	3	4.3263	5.3738	6.2644	7
7	3	4.3263	5.3738	6.2644	7
8	5	5.9203	6.5075	6.8719	7
9	5	5.1940	5.0401	4.6472	4
10	3	3.6937	4.0397	4.1468	4
11	3	3.6937	4.0397	4.1468	4
12	5	5.1940	5.0401	4.6472	4
13	4	3.9175	3.5060	2.8724	2
14	4	3.9175	3.5060	2.8724	2
15	16	11.0347	7.4943	4.6059	2
16	20	12.7098	8.2701	4.9032	2
17	6	4.9782	3.7740	2.4613	1
18	6	4.9782	3.7740	2.4613	1
19	4	3.6232	2.9502	2.0827	1
20	4	3.6232	2.9502	2.0827	1

Table legends: Act – activities: 1-20 as given in Table 1 in Section 2.1; δ_i – severity parameter in [0, 1]; cell contents – frequencies: active times in hours per week (112 active hours in total)

SR1-Table-2: Convergence of distancing strategies in small world social networks

b = 0.30; ρ = 0.20

δ_i = 0.00 \ k =	1	2	3	4	5	6
$<\|x-x^*\|>$	4.5984e-03	5.2092e-04	1.0790e-04	1.2446e-04	7.1508e-05	5.9851e-05
$<\|y-y^*\|>$	4.5943e-03	5.1985e-04	1.0865e-05	6.2008e-06	8.7578e-06	9.0475e-06

δ_i = 0.25 \ k =	1	2	3	4	5	6
$<\|x-x^*\|>$	4.1337e-03	8.4253e-05	1.5355e-04	6.7556e-05	5.5775e-05	5.6646e-05
$<\|y-y^*\|>$	4.1292e-03	7.4418e-05	1.3888e-05	3.6019e-06	7.0071e-06	8.0186e-06

δ_i = 0.50 \ k =	1	2	3	4	5	6
$<\|x-x^*\|>$	3.7795e-03	8.5057e-05	2.1823e-04	6.9527e-05	5.0005e-05	5.6804e-05
$<\|y-y^*\|>$	3.7740e-03	7.1548e-05	1.9280e-05	3.6826e-06	6.8768e-06	7.8958e-06

δ_i = 0.75 \ k =	1	2	3	4	5	6
$<\|x-x^*\|>$	3.9941e-03	1.3703e-04	2.3911e-04	7.1766e-05	5.0727e-05	6.2737e-05
$<\|y-y^*\|>$	3.9891e-03	1.3138e-04	2.1909e-05	4.0323e-06	6.6930e-06	8.8887e-06

δ_i = 1.00 \ k =	1	2	3	4	5	6
$<\|x-x^*\|>$	4.7625e-03	6.3291e-04	1.6536e-04	7.7867e-05	6.7857e-05	6.5584e-05
$<\|y-y^*\|>$	4.7582e-03	6.3230e-04	6.1167e-05	4.2678e-06	8.6237e-06	9.1828e-06

Table legends: k – neighborhood size; δ_i – severity parameter in [0, 1]; x – individual strategy obtained from simulation; y – neighborhood strategy obtained from simulation; $<\|x-x^*\|>$ -- average difference between individual strategy x and equilibrium strategy x*; $<\|y-y^*\|>$ -- average difference between neighborhood strategy y and equilibrium strategy y*

SR1-Table-3: Convergence of distancing strategies in small world social networks

$\delta_i = 0.50$; $\rho = 0.20$

<table>
<tr><th>b = 0.10 \ k =</th><th>1</th><th>2</th><th>3</th><th>4</th><th>5</th><th>6</th></tr>
<tr><td><||x-x*||></td><td>4.5859e-03</td><td>9.6347e-04</td><td>6.5605e-05</td><td>7.3907e-05</td><td>5.0429e-05</td><td>9.3284e-05</td></tr>
<tr><td><||y-y*||></td><td>4.5661e-03</td><td>9.5182e-04</td><td>3.6333e-05</td><td>7.2047e-06</td><td>4.3783e-06</td><td>1.1249e-06</td></tr>
</table>

<table>
<tr><th>b = 0.20 \ k =</th><th>1</th><th>2</th><th>3</th><th>4</th><th>5</th><th>6</th></tr>
<tr><td><||x-x*||></td><td>4.0299e-03</td><td>2.8287e-04</td><td>6.0179e-05</td><td>6.9300e-05</td><td>4.7988e-05</td><td>4.2705e-05</td></tr>
<tr><td><||y-y*||></td><td>4.0204e-03</td><td>2.7849e-04</td><td>7.1976e-06</td><td>4.2490e-06</td><td>4.8773e-06</td><td>6.1604e-06</td></tr>
</table>

<table>
<tr><th>b = 0.30 \ k =</th><th>1</th><th>2</th><th>3</th><th>4</th><th>5</th><th>6</th></tr>
<tr><td><||x-x*||></td><td>3.7795e-03</td><td>8.5057e-05</td><td>2.1823e-04</td><td>6.9527e-05</td><td>5.0005e-05</td><td>5.6804e-05</td></tr>
<tr><td><||y-y*||></td><td>3.7740e-03</td><td>7.1548e-05</td><td>1.9280e-05</td><td>3.6826e-06</td><td>6.8768e-06</td><td>7.8958e-06</td></tr>
</table>

<table>
<tr><th>b = 0.40 \ k =</th><th>1</th><th>2</th><th>3</th><th>4</th><th>5</th><th>6</th></tr>
<tr><td><||x-x*||></td><td>3.6936e-03</td><td>5.6663e-05</td><td>2.9138e-04</td><td>6.9256e-05</td><td>6.4721e-05</td><td>6.7787e-05</td></tr>
<tr><td><||y-y*||></td><td>3.6907e-03</td><td>2.3668e-05</td><td>2.2495e-05</td><td>5.3908e-06</td><td>8.8238e-06</td><td>8.8134e-06</td></tr>
</table>

<table>
<tr><th>b = 0.50 \ k =</th><th>1</th><th>2</th><th>3</th><th>4</th><th>5</th><th>6</th></tr>
<tr><td><||x-x*||></td><td>3.6267e-03</td><td>6.3502e-05</td><td>2.9609e-04</td><td>7.6744e-05</td><td>7.5655e-05</td><td>7.5522e-05</td></tr>
<tr><td><||y-y*||></td><td>3.6246e-03</td><td>1.5391e-05</td><td>2.1485e-05</td><td>7.8607e-06</td><td>1.0026e-05</td><td>9.2847e-06</td></tr>
</table>

Table legends: k – neighborhood size; b – randomness parameter for the network; x – individual strategy obtained from simulation; y – neighborhood strategy obtained from simulation; <||x-x*||> -- average difference between individual strategy x and equilibrium strategy x*; <||y-y*||> -- average difference between neighborhood strategy y and equilibrium strategy y*

SR1-Table-4: Convergence of distancing strategies in small world social networks

δ_i = 0.50: b = 0.30:

<table>
<tr><th>ρ = 0.00 \ k =</th><th>1</th><th>2</th><th>3</th><th>4</th><th>5</th><th>6</th></tr>
<tr><td><||x-x*||></td><td>7.2551e-04</td><td>1.0668e-04</td><td>3.9351e-04</td><td>2.9802e-04</td><td>7.3131e-05</td><td>5.6947e-05</td></tr>
<tr><td><||y-y*||></td><td>6.3989e-04</td><td>2.1839e-05</td><td>3.2507e-05</td><td>1.1306e-05</td><td>5.9527e-06</td><td>8.5252e-06</td></tr>
</table>

<table>
<tr><th>ρ = 0.10 \ k =</th><th>1</th><th>2</th><th>3</th><th>4</th><th>5</th><th>6</th></tr>
<tr><td><||x-x*||></td><td>1.1797e-03</td><td>6.6783e-05</td><td>3.0992e-04</td><td>1.2071e-04</td><td>6.2364e-05</td><td>5.0019e-05</td></tr>
<tr><td><||y-y*||></td><td>1.1655e-03</td><td>1.5032e-05</td><td>2.6263e-05</td><td>5.4969e-06</td><td>7.6969e-06</td><td>7.6941e-06</td></tr>
</table>

<table>
<tr><th>ρ = 0.20 \ k =</th><th>1</th><th>2</th><th>3</th><th>4</th><th>5</th><th>6</th></tr>
<tr><td><||x-x*||></td><td>3.7795e-03</td><td>8.5057e-05</td><td>2.1823e-04</td><td>6.9527e-05</td><td>5.0005e-05</td><td>5.6804e-05</td></tr>
<tr><td><||y-y*||></td><td>3.7740e-03</td><td>7.1548e-05</td><td>1.9280e-05</td><td>3.6826e-06</td><td>6.8768e-06</td><td>7.8958e-06</td></tr>
</table>

<table>
<tr><th>ρ = 0.30 \ k =</th><th>1</th><th>2</th><th>3</th><th>4</th><th>5</th><th>6</th></tr>
<tr><td><||x-x*||></td><td>1.0191e-02</td><td>5.3876e-03</td><td>4.4098e-03</td><td>4.1806e-03</td><td>4.0781e-03</td><td>4.0422e-03</td></tr>
<tr><td><||y-y*||></td><td>1.0189e-02</td><td>5.3880e-03</td><td>4.4101e-03</td><td>4.1806e-03</td><td>4.0781e-03</td><td>4.0422e-03</td></tr>
</table>

<table>
<tr><th>ρ = 0.40 \ k =</th><th>1</th><th>2</th><th>3</th><th>4</th><th>5</th><th>6</th></tr>
<tr><td><||x-x*||></td><td>2.0797e-02</td><td>1.7003e-02</td><td>1.6205e-02</td><td>1.5897e-02</td><td>1.5764e-02</td><td>1.5710e-02</td></tr>
<tr><td><||y-y*||></td><td>2.0796e-02</td><td>1.7003e-02</td><td>1.6205e-02</td><td>1.5898e-02</td><td>1.5764e-02</td><td>1.5710e-02</td></tr>
</table>

Table legends: k – neighborhood size; ρ – perturbation rate for generating initial strategies; x – individual strategy obtained from simulation; y – neighborhood strategy obtained from simulation; <||x-x*||> -- average difference between individual strategy x and equilibrium strategy x*; <||y-y*||> -- average difference between neighborhood strategy y and equilibrium strategy y*

SR1-Figure-1: Convergence of distancing strategies in small world social networks

$\delta_i = 0.50$, $b = 0.30$, $\rho = 0.20$, $k = 1, 2$

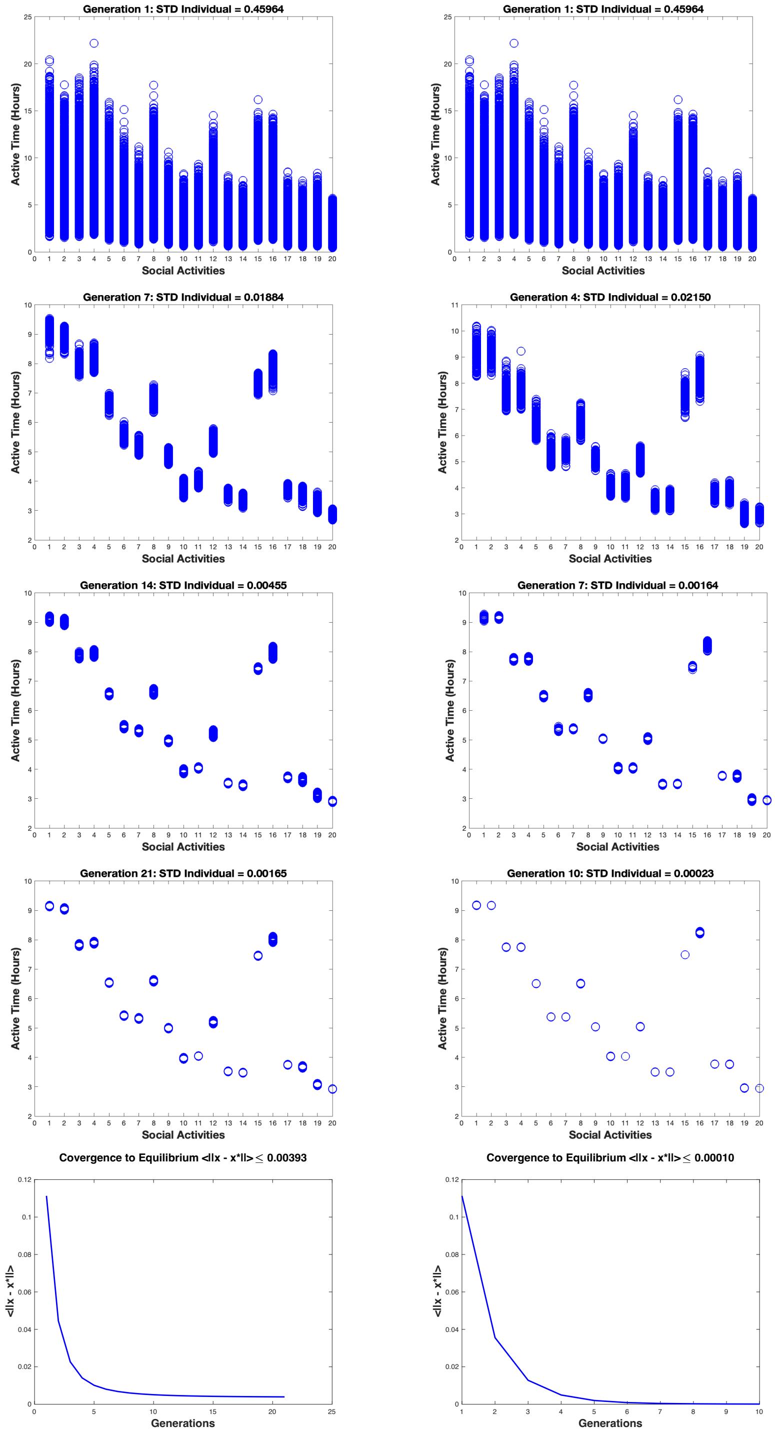

SR1-Figure-2: Convergence of distancing strategies in small world social networks

$\delta_i = 0.50$, $b = 0.30$, $\rho = 0.20$, $k = 3, 4$

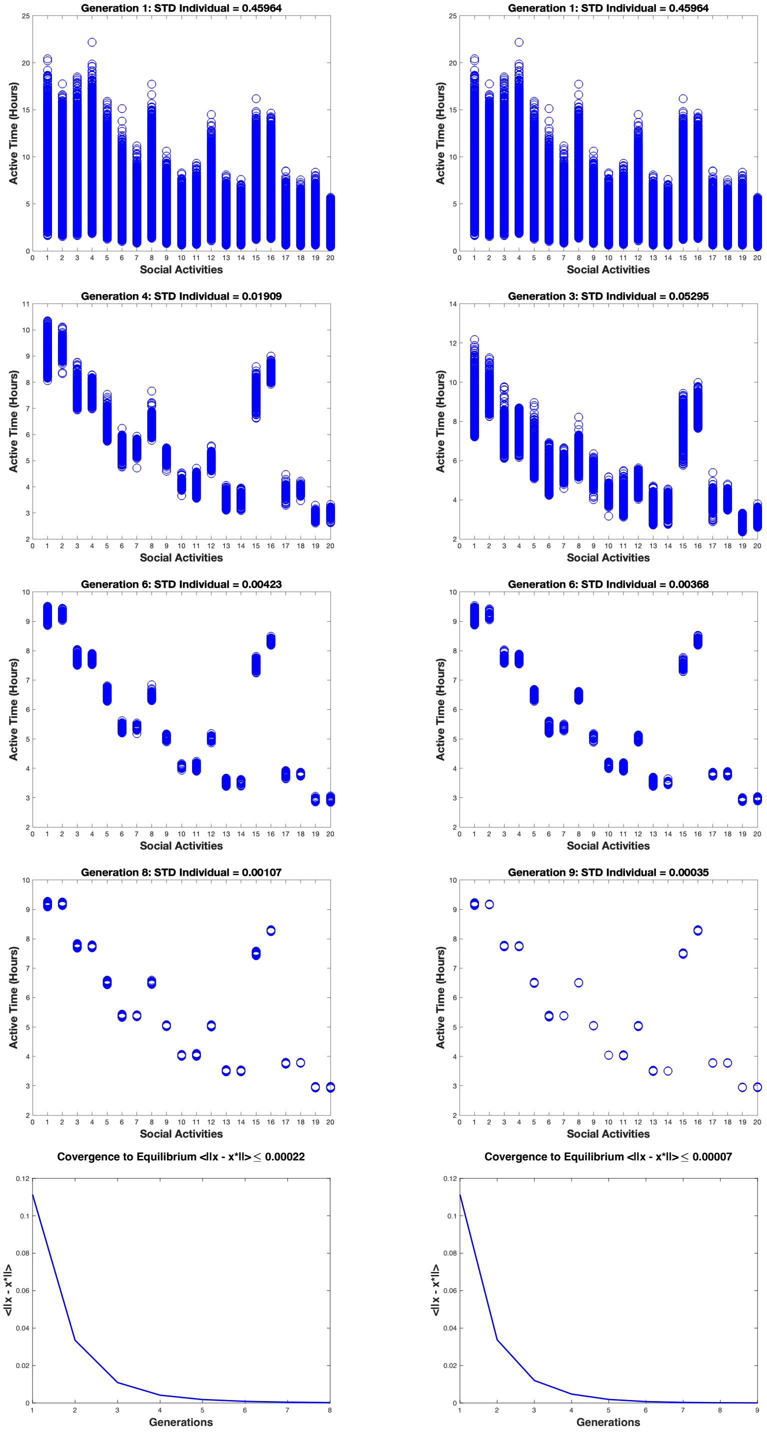

SR1-Figure-3: Convergence of distancing strategies in small world social networks

$\delta_i = 0.50$, $b = 0.30$, $\rho = 0.20$, $k = 5, 6$

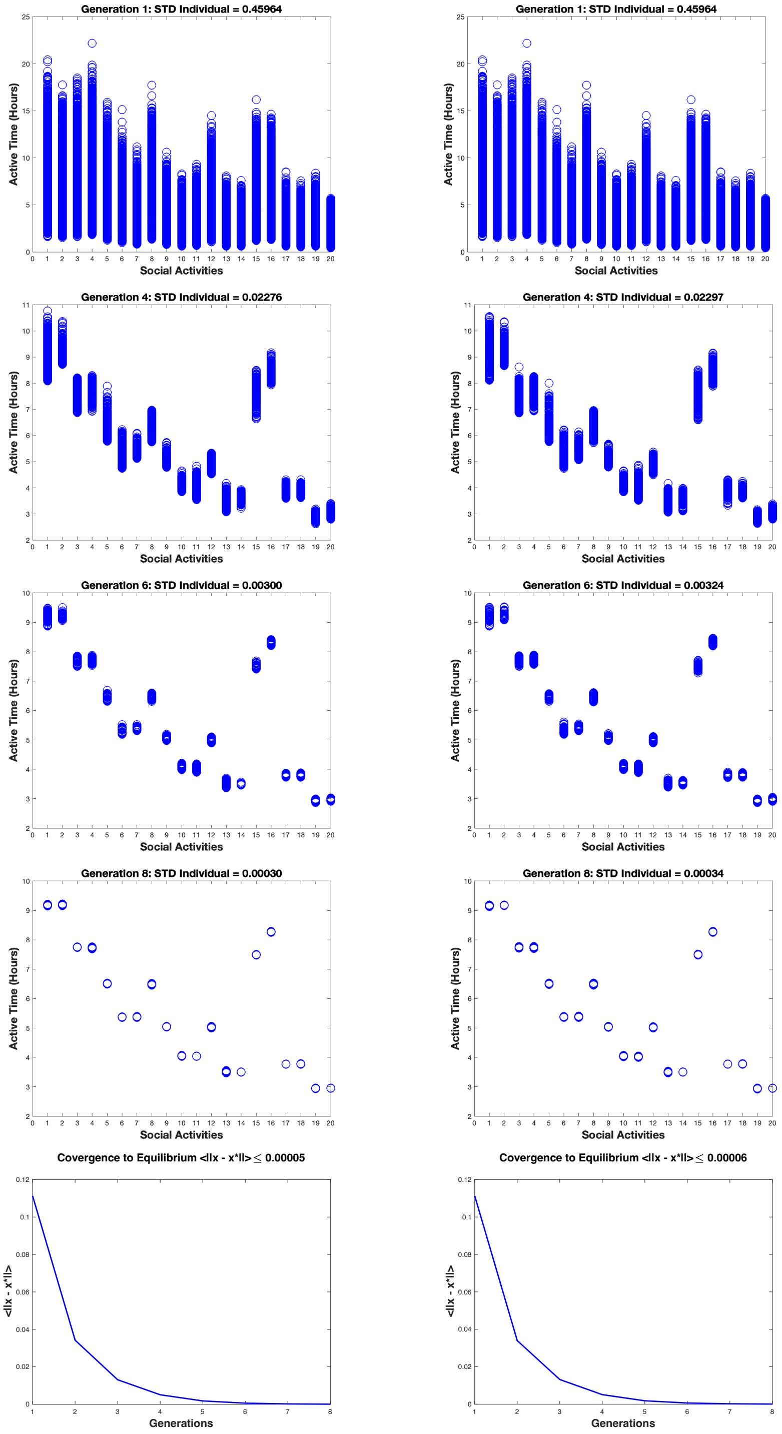

Supplement: S1 Text — (PDF) [file pone.0293489.s001.pdf]
