# Supplementary material for: Beyond six feet: The collective behavior of social distancing
Source: PLoS One. 2024 Sep 13;19(9):e0293489. doi: 10.1371/journal.pone.0293489 (PMC11398703; doi:10.1371/journal.pone.0293489)

Simulation Results 2: In Heterogeneous Populations

Results in SR2-Table-1:

The table contains the equilibrium strategies for different population groups with different values for the severity parameters. Note that each of these equilibrium strategies is equivalent to the one when the game is played with only the corresponding population group. Therefore, assume that there are n activities, and the activities are independent. Let $x = \{x_i : i = 1, .., n\}$ be the distancing strategy of an individual and $y = \{y_i : i = 1, …, n\}$ the average strategy of the population. Let $p_i(y) = w_i\sigma_i(y_i)$ be the potential distancing risk at activity i for a population group, where w_i is the risk factor of activity i for this group. Then, the equilibrium strategy of this individual in this group can be obtained with a formula, $x_i^* = \sigma_i^{-1}(\lambda/w_i)$ for all i, where λ is a constant such that $1 = \Sigma_i \sigma_i^{-1}(\lambda/w_i)$ (details in Methods – Distancing in heterogeneous populations).

Results in SR2-Table-2:

This table shows the convergence of the distancing strategies to their equilibrium strategies in heterogeneous populations. The simulation is done with the population distributed over a small world social network. The network parameter m is fixed to 2000, K to 6, but b varied from 0.1 to 0.2, 0.3, 0.4, 0.5. The game is also simulated for different neighborhood sizes: k = 1, 2, 3, 4, 5, and 6. The population is divided into four groups, g_1, g_2, g_3, g_4. The severity parameter δ_i is set to 0.00 for g_1, 0.25 for g_2, 0.75 for g_3, and 1.00 for g_4. Shown in the table are the average Euclidean norms of the differences between individual strategies and their corresponding group equilibrium strategies. In a heterogeneous population, individuals in different population groups may have different equilibrium strategies as given in SR2-Table-1.

The simulation is started with every individual assigned an initial strategy x randomly generated around its group equilibrium strategy x^*. More specifically, for every individual, and for every activity i, x_i^* is first perturbed randomly by 20%; an initial frequency x_i is then generated randomly within 100% of deviation from the perturbed value of x_i^*. The simulation then proceeds and terminates, in most cases, in less than 20 generations, when either the average difference between every individual strategy and its corresponding group equilibrium strategy is small enough or no strategy is improved in a few consecutive generations.

The simulation is repeated for five times with five different sets of randomly generated initial strategies for all the individuals. The results in the table are the average outcomes. The results in SR2-Table-3 are obtained with the same simulation procedure as described above except that δ_i is set to 0.25 for g_1, 0.25 for g_2, 0.75 for g_3, and 0.75 for g_4.

The results in SR2-Table-2 and SR2-Table-3 show that the individual strategies converge to their group equilibrium strategies in most cases for different levels of randomness of the network. When the neighborhood size is small with k = 1, the simulation fails to converge. When k = 2,

the simulation still sees some difficulties to converge: the accuracy is not as high. However, when $k \geq 3$, the simulation is able to converge well.

Results in SR2-Figure-1:

This figure contains the snapshots from the simulation for the distancing game in a heterogeneous population with four different population groups, g_1, g_2, g_3, g_4. The balancing parameter δ_i is set to 0.00 for g_1, 0.25 for g_2, 0.75 for g_3, and 1.00 for g_4. The parameters for the network are m = 2000, K = 6, b = 0.3. There are two columns of plots in the figure. The first one is from the simulation with neighborhood size k = 1. The second one is from the simulation with neighborhood size k = 2.

In each column, the first four plots show the changes of the individual strategies in four different generations. In each of the plots, along the x-axis are 20 CASA activities. Over each activity, there are 2000 circles corresponding to the participating frequencies of the 2000 individuals in this activity. The circles are color coded for different population groups, red for g_1, magenta for g_2, cyan for g_3, and blue for g_4. The average participating frequencies in the activities in the whole population are marked by the stars. Along the y-axis are the participating frequencies for the activities represented by the active times in hours per week (112 active hours in total).

The last plot in each column shows the changes of the average difference between every individual strategy and its group equilibrium strategy. As shown in the first column, for k = 1, the individual strategies are not changed much over generations. The average difference between every individual strategy and its group equilibrium strategy is not reduced much either after 14 generations when the simulation is terminated.

However, when k = 2, as shown in the second column, the individual strategies converge to their group equilibrium strategies, although not in high accuracy. The first plot shows the initial strategies at the 1st generation when they appear to be quite random; the second plot shows the strategies in the 5th generation when they start converging; the third plot shows the strategies in the 10th generation when they almost converge to their equilibrium positions; the fourth plot shows the strategies in the 14th generation when they are not making further improvements, and the simulation is terminated. As shown in the last plot, the average difference between every individual strategy and its group equilibrium strategy is decreased as the generation increases and is eventually reduced to < 0.00604.

The results in SR2-Figure-2 and SR2-Figure-3 are produced in the same way as those in SR2-Figure-1 except that SR2-Figure-2 shows the results for k = 3, 4 and SR2-Figure-3 for k = 5, 6. The results for k = 3, 4, 5, 6 are all similar to or better than those for k = 2, showing that the distancing strategies converge to their group equilibrium strategies for all neighborhood sizes greater than or equal to 2.

SR2-Table-1. Group equilibrium strategies x* with different severity parameters

Act \ δ_i =	0.00	0.25	0.75	1.00
1	5	7.1175	11.3984	14
2	5	7.1175	11.3984	14
3	3	5.3571	10.5003	14
4	3	5.3571	10.5003	14
5	5	5.9203	6.8719	7
6	3	4.3263	6.2644	7
7	3	4.3263	6.2644	7
8	5	5.9203	6.8719	7
9	5	5.1940	4.6472	4
10	3	3.6937	4.1468	4
11	3	3.6937	4.1468	4
12	5	5.1940	4.6472	4
13	4	3.9175	2.8724	2
14	4	3.9175	2.8724	2
15	16	11.0347	4.6059	2
16	20	12.7098	4.9032	2
17	6	4.9782	2.4613	1
18	6	4.9782	2.4613	1
19	4	3.6232	2.0827	1
20	4	3.6232	2.0827	1

Table legends: Act – activities: 1-20 as given in Table 1 in Section 2.1; δ_i – severity parameter in [0, 1]; cell contents – frequencies: active times in hours per week (112 active hours in total)

SR2-Table-2: Convergence of distancing strategies in heterogeneous populations

g_1: δ_i = 0.00, g_2: δ_i = 0.25, g_3: δ_i = 0.75, g_4: δ_i = 1.00; perturbation: ρ = 0.20

b = 0.10 \ k =	1	2	3	4	5	6
g_1: $\langle \lVert x-x^* \rVert \rangle$	1.0932e-01	1.1246e-02	4.4307e-03	1.4245e-03	3.7901e-04	2.3518e-04
g_2: $\langle \lVert x-x^* \rVert \rangle$	1.0826e-01	9.1976e-03	3.9045e-03	1.0101e-03	2.1234e-04	1.2118e-04
g_3: $\langle \lVert x-x^* \rVert \rangle$	1.0636e-01	9.6739e-03	3.7493e-03	6.2948e-04	1.4865e-04	1.1987e-04
g_4: $\langle \lVert x-x^* \rVert \rangle$	1.1953e-01	1.0060e-02	3.6245e-03	1.5032e-03	9.1589e-04	6.7787e-04

b = 0.20 \ k =	1	2	3	4	5	6
g_1: $\langle \lVert x-x^* \rVert \rangle$	1.0405e-01	8.4517e-03	2.1354e-03	3.6794e-04	1.7168e-04	1.3033e-04
g_2: $\langle \lVert x-x^* \rVert \rangle$	1.0347e-01	7.1092e-03	1.8763e-03	2.1091e-04	1.0697e-04	9.0090e-05
g_3: $\langle \lVert x-x^* \rVert \rangle$	1.0162e-01	7.3297e-03	1.6649e-03	1.4482e-04	1.0747e-04	8.9172e-05
g_4: $\langle \lVert x-x^* \rVert \rangle$	1.1106e-01	7.4078e-03	1.8820e-03	9.1207e-04	6.3626e-04	5.3305e-04

b = 0.30 \ k =	1	2	3	4	5	6
g_1: $\langle \lVert x-x^* \rVert \rangle$	1.0176e-01	6.9870e-03	1.3580e-03	1.9419e-04	1.3986e-04	1.0980e-04
g_2: $\langle \lVert x-x^* \rVert \rangle$	9.7123e-02	5.6781e-03	9.1068e-04	1.4413e-04	9.1948e-05	8.4924e-05
g_3: $\langle \lVert x-x^* \rVert \rangle$	9.7701e-02	6.1939e-03	6.4404e-04	1.2688e-04	9.2106e-05	8.4280e-05
g_4: $\langle \lVert x-x^* \rVert \rangle$	1.0704e-01	5.9089e-03	1.5397e-03	7.4641e-04	5.4213e-04	5.0688e-04

b = 0.40 \ k =	1	2	3	4	5	6
g_1: $\langle \lVert x-x^* \rVert \rangle$	9.7531e-02	5.8834e-03	8.5146e-04	2.1741e-04	1.3408e-04	1.0844e-04
g_2: $\langle \lVert x-x^* \rVert \rangle$	9.2935e-02	4.8577e-03	5.5718e-04	1.1610e-04	8.4403e-05	8.4376e-05
g_3: $\langle \lVert x-x^* \rVert \rangle$	9.4490e-02	5.0101e-03	3.0507e-04	1.1362e-04	8.4767e-05	8.3678e-05
g_4: $\langle \lVert x-x^* \rVert \rangle$	1.0502e-01	4.7595e-03	1.2716e-03	6.2248e-04	4.9747e-04	5.0220e-04

b = 0.50 \ k =	1	2	3	4	5	6
g_1: $\langle \lVert x-x^* \rVert \rangle$	9.6418e-02	5.1139e-03	5.0018e-04	1.9518e-04	1.2185e-04	1.0443e-04
g_2: $\langle \lVert x-x^* \rVert \rangle$	9.2712e-02	4.3767e-03	3.3474e-04	1.1521e-04	8.4406e-05	8.2947e-05
g_3: $\langle \lVert x-x^* \rVert \rangle$	9.4258e-02	4.4150e-03	1.8121e-04	1.1233e-04	8.4545e-05	8.2819e-05
g_4: $\langle \lVert x-x^* \rVert \rangle$	1.0130e-01	4.3969e-03	1.1011e-03	5.9476e-04	4.9921e-04	4.9381e-04

Table legends: k – neighborhood size; b – randomness parameter for the network; x – individual strategy obtained from simulation; $\langle \lVert x-x^* \rVert \rangle$ -- average difference between individual strategy x and group equilibrium strategy x*

SR2-Table-3: Convergence of distancing strategies in heterogeneous populations

g_1: $\delta_i = 0.25$, g_2: $\delta_i = 0.25$, g_3: $\delta_i = 0.75$, g_4: $\delta_i = 0.75$; perturbation: $\rho = 0.20$

b = 0.10 \ k =	1	2	3	4	5	6
g_1: <||x-x*||>	1.0139e-01	1.0775e-02	4.4525e-03	7.5889e-04	6,4174e-05	5.6171e-05
g_2: <||x-x*||>	1.0824e-01	9.2497e-03	3.8608e-03	9.4864e-04	1.1903e-04	5.8957e-05
g_3: <||x-x*||>	1.0633e-01	9.9404e-03	3.8499e-03	6.0474e-04	5.9797e-05	5.7511e-05
g_4: <||x-x*||>	1.0759e-01	9.6775e-03	3.9014e-03	1.1760e-03	2.7128e-04	1.3024e-04

b = 0.20 \ k =	1	2	3	4	5	6
g_1: <||x-x*||>	9.6572e-02	8.3284e-03	1.5213e-03	6.5712e-05	7.1618e-05	5.2045e-05
g_2: <||x-x*||>	1.0345e-01	7.0665e-03	1.7938e-03	1.2402e-04	7.7662e-05	6.0028e-05
g_3: <||x-x*||>	1.0146e-01	7.5267e-03	1.6161e-03	6.3815e-05	7.2088e-05	5.3875e-05
g_4: <||x-x*||>	1.0048e-01	7.2310e-03	1.7833e-03	2.9014e-04	1.3891e-04	1.2429e-04

b = 0.30 \ k =	1	2	3	4	5	6
g_1: <||x-x*||>	9.4553e-02	7.0325e-03	8.0877e-04	4.5575e-05	4.2141e-05	4.7754e-05
g_2: <||x-x*||>	9.6844e-02	5.5995e-03	6.5724e-04	5.6440e-05	4.2696e-05	4.6619e-05
g_3: <||x-x*||>	9.7650e-02	6.3272e-03	1.0793e-03	4.7796e-05	9.5426e-05	9.9920e-05
g_4: <||x-x*||>	9.6901e-02	5.8689e-03	1.4094e-04	9.9504e-05	1.0198e-05	1.0904e-05

b = 0.40 \ k =	1	2	3	4	5	6
g_1: <||x-x*||>	9.0682e-02	5.8676e-03	2.3590e-04	6.6869e-05	4.3305e-05	4.3035e-05
g_2: <||x-x*||>	9.2576e-02	4.8890e-03	4.2212e-04	7.4091e-05	4.5754e-05	4.8204e-05
g_3: <||x-x*||>	9.4276e-02	5.1815e-03	2.5009e-04	6.1162e-05	4.8879e-05	5.2153e-05
g_4: <||x-x*||>	9.5355e-02	5.0218e-03	7.1215e-04	1.2017e-04	1.0091e-04	1.0271e-04

b = 0.50 \ k =	1	2	3	4	5	6
g_1: <||x-x*||>	8.9597e-02	5.0261e-03	1.3342e-04	5.9764e-05	4.3356e-05	4.3841e-05
g_2: <||x-x*||>	9.2330e-02	4.3327e-03	2.7500e-04	7.9457e-05	4.8281e-05	4.8097e-05
g_3: <||x-x*||>	9.4137e-02	4.6035e-03	1.4586e-04	5.5941e-05	5.0573e-05	5.5970e-05
g_4: <||x-x*||>	9.2205e-02	4.5547e-03	5.3461e-04	9.5516e-05	8.9281e-05	1.0043e-04

Table legends: k – neighborhood size; b – randomness parameter for the network; x – individual strategy obtained from simulation; <||x-x*||> -- average difference between individual strategy x and group equilibrium strategy x*

SR2-Figure-1: Convergence of distancing strategies in heterogeneous populations

g_1 – red, g_2 – magenta, g_3 – cyan, g_4 – blue, all – stars; g_1: δ_i = 0.00; g_2: δ_i = 0.25; g_3: δ_i = 0.75; g_4: δ_i = 1.00; randomness of network: b = 0.30; neighborhood size: k = 1, 2

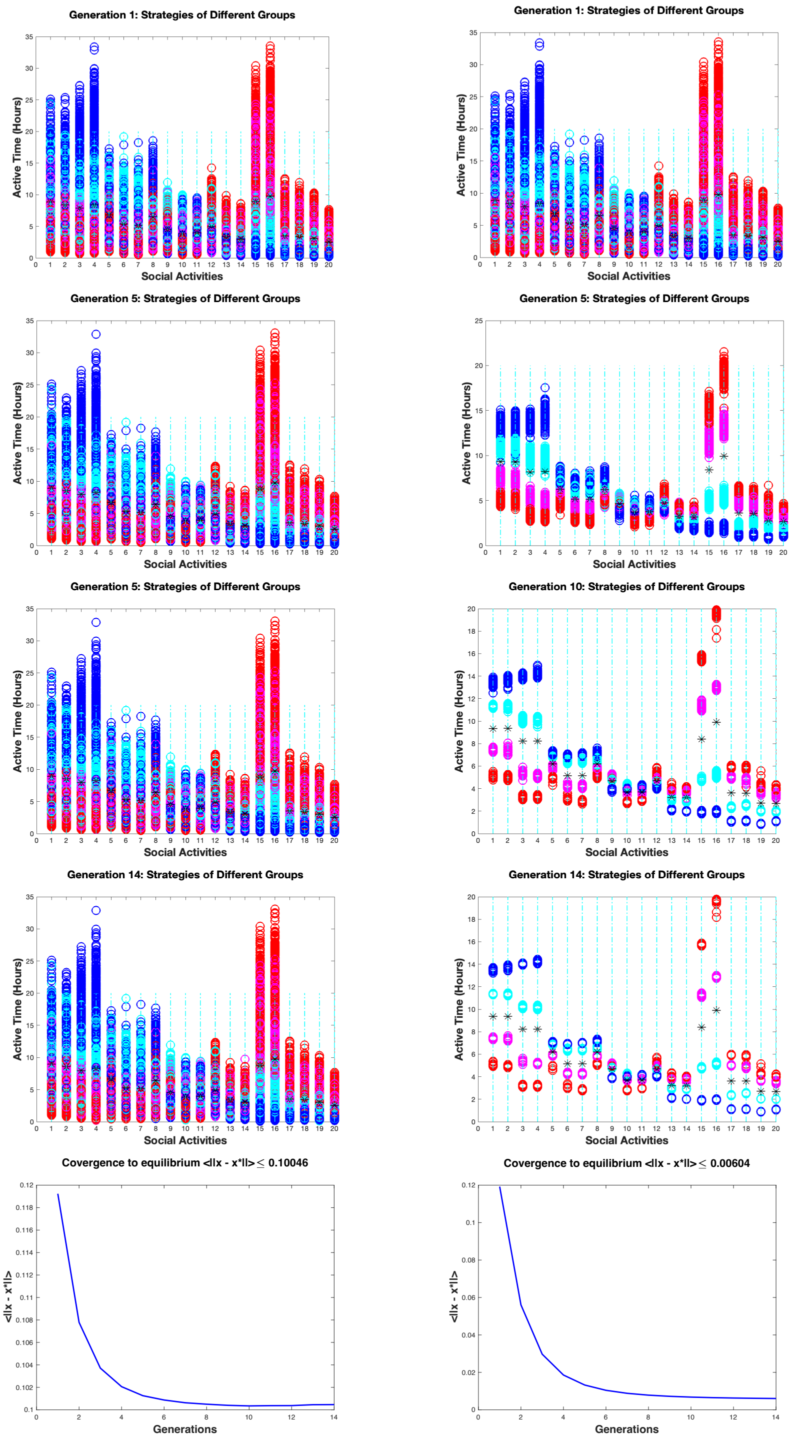

SR2-Figure-2: Convergence of distancing strategies in heterogeneous populations

g_1 – red, g_2 – magenta, g_3 – cyan, g_4 – blue, all – stars; g_1: $\delta_i = 0.00$; g_2: $\delta_i = 0.25$; g_3: $\delta_i = 0.75$; g_4: $\delta_i = 1.00$; randomness of network: $b = 0.30$; neighborhood size: $k = 3, 4$

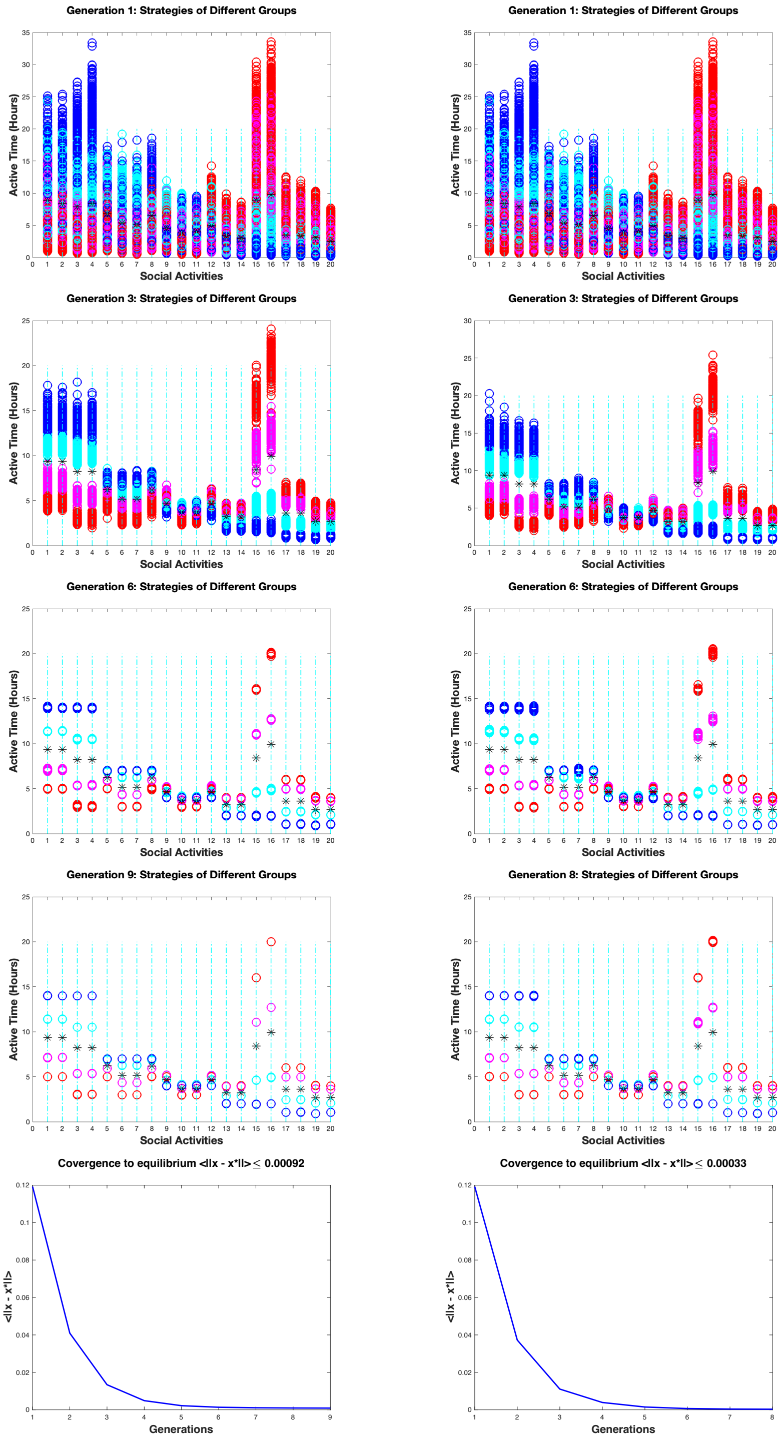

SR2-Figure-3: Convergence of distancing strategies in heterogeneous populations

g_1 – red, g_2 – magenta, g_3 – cyan, g_4 – blue, all – stars; g_1: $\delta_i = 0.00$; g_2: $\delta_i = 0.25$; g_3: $\delta_i = 0.75$; g_4: $\delta_i = 1.00$; randomness of network: b = 0.30; neighborhood size: k = 5, 6

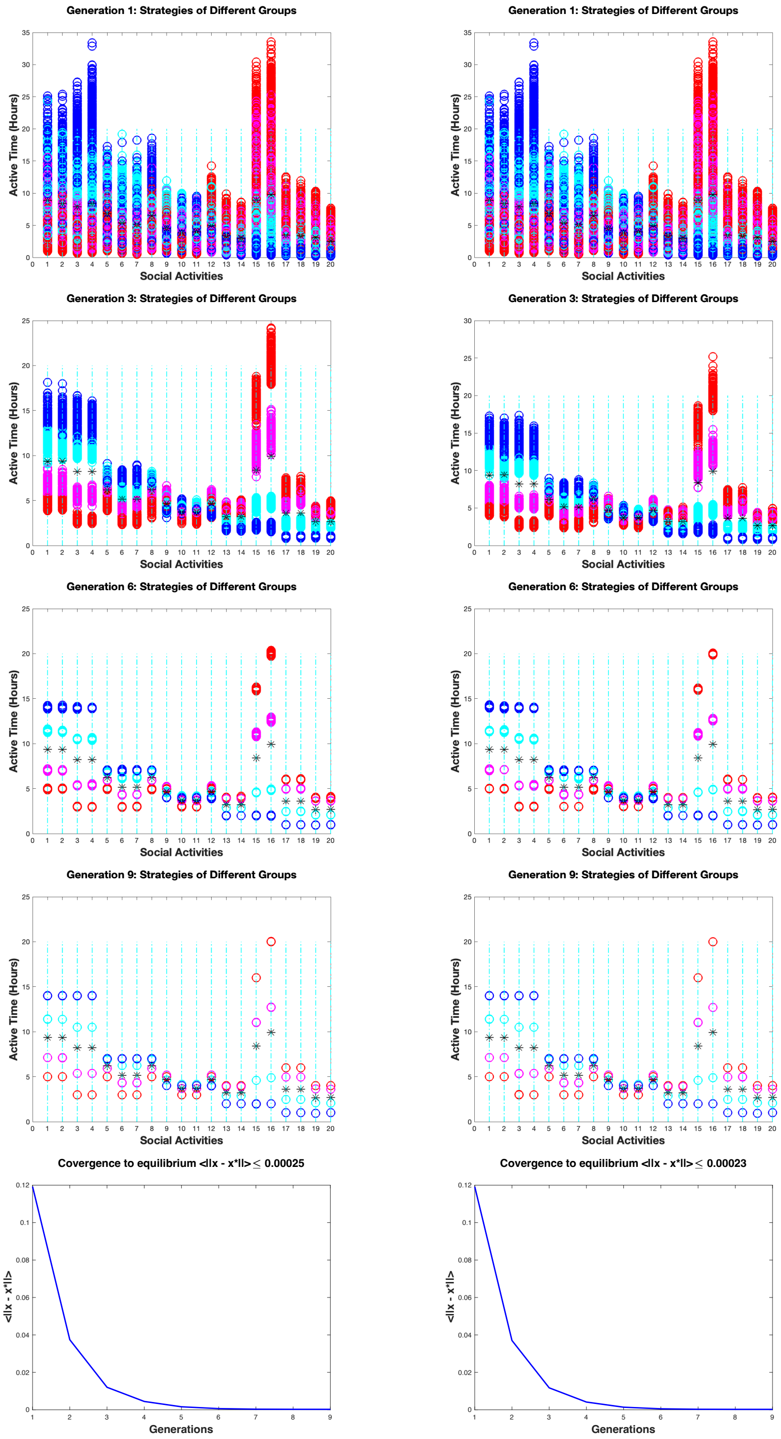

Supplement: S2 Text — (PDF) [file pone.0293489.s002.pdf]
