# Supplementary material for: Beyond six feet: The collective behavior of social distancing
Source: PLoS One. 2024 Sep 13;19(9):e0293489. doi: 10.1371/journal.pone.0293489 (PMC11398703; doi:10.1371/journal.pone.0293489)

Simulation Results 3: By Following the Leaders

Results in SR3-Table-1:

This table shows the convergence of the distancing strategies to their equilibrium strategies by following the leaders. The simulation is done with the population distributed over a small world social network. The network parameter m is fixed to 2000, K to 6, and b to 0.3. The game is simulated with different percentages of individuals assigned randomly as leaders, i.e., e = 10%, 20%, 30%, 40%, 50%, and with different neighborhood sizes, i.e., k = 1, 2, 3, 4, 5, 6. The population is divided into four groups, g_1, g_2, g_3, g_4. The balancing parameter δ_i is set to 0.00 for g_1, 0.25 for g_2, 0.75 for g_3, and 1.00 for g_4. Shown in the table are the average Euclidean norms of the differences between individual strategies and their corresponding group equilibrium strategies.

The simulation is started with every individual assigned an initial strategy x randomly generated around its group equilibrium strategy x*. More specifically, for every individual, and for every activity i, x_i* is first perturbed randomly by 20%; an initial frequency x_i is then generated randomly within 100% of deviation from the perturbed value of x_i*.

The simulation proceeds with the leaders making regular distancing decisions while the followers just copy the strategies from their group leaders in their neighborhoods. If an individual cannot find a group leader among his/her closest neighbors, he/she just follows the crowd, i.e., takes the average group strategy in the neighborhood. The simulation terminates, in most cases, in less than 20 generations, when either the average difference between every individual strategy and its corresponding group equilibrium strategy is small enough or no strategy is improved in a few consecutive generations. The simulation is repeated for five times with different sets of randomly generated initial strategies for all the individuals. The results in SR3-Table-1 are the average outcomes.

The results in SR3-Table-2 are generated with the same simulation procedure except when an individual cannot find a group leader among his/her closest neighbors, he/she makes his/her own distancing decision. The results are quite different. As shown in SR3-Table-1, when there are fewer leaders in the population with $e \leq 20\%$ and the neighborhood size is smaller with $k \leq 3$, a follower would have a hard time to find a group leader among his/her closest neighbors, and he/she must follow the crowd, which is not as good as following a leader, and therefore, the game would not converge well.

However, as shown in SR3-Table-2, with a self-determination strategy, the convergence of the game remains to be accurate even when there are fewer leaders ($e \leq 20\%$) in the population and the neighborhood size is small (k = 2, 3), showing that making own decisions are more reliable in general than following the crowd when there are fewer leaders.

Results in SR3-Figure-1:

This figure contains the snapshots from the simulation of a distancing game led by 30% of individuals in the population. A follow-the-crowd strategy is used when a group leader cannot be found. The balancing parameter δ_i is 0.00 for g_1, 0.25 for g_2, 0.75 for g_3, and 1.00 for g_4. The parameters for the network are m = 2000, K = 6, and b = 0.3. There are two columns of plots. The first one is from the simulation with neighborhood size k = 1. The second one is from the simulation with neighborhood size k = 2.

In each column, the first four plots show the changes of the individual strategies in four different generations. In each of the plots, along the x-axis are 20 CASA activities. Over each activity, there are 2000 circles corresponding to the participating frequencies of the 2000 individuals in this activity. The circles are color coded for different population groups, red for g_1, magenta for g_2, cyan for g_3, and blue for g_4. The average participating frequencies in the activities in the whole population are marked by the stars. Along the y-axis are the participating frequencies for the activities represented by the active times in hours per week (112 active hours in total).

The last plot in each column shows the changes of the average difference between the individual strategy and the group equilibrium strategy. As shown in the first column, for k = 1, the individual strategies are not changed much over generations. The average difference between every individual strategy and the group equilibrium strategy is not reduced much either after a few generations when the simulation is terminated.

When k = 2, as shown in the second column, the individual strategies converge better but not as accurate as expected and take many generations. The first plot shows the initial strategies at the 1st generation when they appear to be quite random; the second plot shows the strategies in the 6th generation when they start converging; the third plot shows the strategies in the 12th generation when they converge toward their equilibrium positions; the fourth plot shows the strategies in the 19th generation when they are not improved further, and the simulation is terminated. As shown in the last plot, the average difference between every individual strategy and the group equilibrium strategy is eventually reduced to < 0.01393.

Results in SR3-Figure-2 and SR3-Figure-3 are produced in the same way as those in SR3-Figure-1 except that SR3-Figure-2 is for k = 3, 4 and SR3-Figure-3 for k = 5, 6. The results for k = 3 as shown in SR3-Figure-2 are much better than those for k = 2. For k = 4, 5, 6, the results are about the same: The simulation takes only 10 generations to converge, and the accuracy of the convergence is more acceptable with the average difference between every individual strategy and its group equilibrium strategy around 0.006 in the end.

SR3-Table-1: Convergence of distancing strategies by following the leaders

g_1: $\delta_i = 0.00$, g_2: $\delta_i = 0.25$, g_3: $\delta_i = 0.75$, g_4: $\delta_i = 1.00$; network randomness: b = 0.30; perturbation: $\rho = 0.20$

e = 0.10 \ k =	1	2	3	4	5	6
g_1: $\langle \lVert x-x^* \rVert \rangle$	1.0685e-01	1.9527e-02	1.6697e-02	1.4059e-02	1.2925e-02	1.2493e-02
g_2: $\langle \lVert x-x^* \rVert \rangle$	9.8185e-02	1.7128e-02	1.3564e-02	1.1201e-02	1.0772e-02	1.0942e-02
g_3: $\langle \lVert x-x^* \rVert \rangle$	1.0587e-01	2.3613e-02	2.0940e-02	1.8103e-02	1.6875e-02	1.6809e-02
g_4: $\langle \lVert x-x^* \rVert \rangle$	1.1522e-01	1.7401e-02	1.4089e-02	1.1006e-02	1.0667e-02	1.0808e-02

e = 0.20 \ k =	1	2	3	4	5	6
g_1: $\langle \lVert x-x^* \rVert \rangle$	1.0950e-01	1.5365e-02	1.0632e-02	7.9932e-03	7.5424e-03	7.1890e-03
g_2: $\langle \lVert x-x^* \rVert \rangle$	9.9333e-02	1.5025e-02	1.0463e-02	8.1841e-03	7.8725e-03	7.9342e-03
g_3: $\langle \lVert x-x^* \rVert \rangle$	1.0666e-01	1.9783e-02	1.5410e-02	1.1393e-02	1.0186e-02	1.0153e-02
g_4: $\langle \lVert x-x^* \rVert \rangle$	1.1590e-01	1.5428e-02	1.0992e-02	7.4395e-03	7.1571e-03	7.4008e-03

e = 0.30 \ k =	1	2	3	4	5	6
g_1: $\langle \lVert x-x^* \rVert \rangle$	1.1035e-01	1.2262e-02	7.2883e-03	5.1538e-03	4.4021e-03	4.1314e-03
g_2: $\langle \lVert x-x^* \rVert \rangle$	9.8561e-02	1.2740e-02	8.2715e-03	6.3081e-03	6.1232e-03	6.1951e-03
g_3: $\langle \lVert x-x^* \rVert \rangle$	1.0603e-01	1.7706e-02	1.2393e-02	8.4837e-03	7.2615e-03	7.0887e-03
g_4: $\langle \lVert x-x^* \rVert \rangle$	1.1620e-01	1.3955e-02	8.9342e-03	5.7216e-03	5.4282e-03	5.6152e-03

e = 0.40 \ k =	1	2	3	4	5	6
g_1: $\langle \lVert x-x^* \rVert \rangle$	1.0923e-01	1.0312e-02	5.0961e-03	3.0325e-03	2.3869e-03	2.2398e-03
g_2: $\langle \lVert x-x^* \rVert \rangle$	9.8548e-02	1.0220e-02	5.6613e-03	4.2217e-03	4.1480e-03	4.2412e-03
g_3: $\langle \lVert x-x^* \rVert \rangle$	1.0645e-01	1.6359e-02	1.0566e-02	6.5855e-03	5.3676e-03	5.2561e-03
g_4: $\langle \lVert x-x^* \rVert \rangle$	1.1579e-01	1.2957e-02	7.2468e-03	4.1764e-03	4.0169e-03	4.0130e-03

e = 0.50 \ k =	1	2	3	4	5	6
g_1: $\langle \lVert x-x^* \rVert \rangle$	1.0904e-01	8.9166e-03	4.0525e-03	2.2166e-03	1.5663e-03	1.4094e-03
g_2: $\langle \lVert x-x^* \rVert \rangle$	9.8095e-02	9.2040e-03	4.6434e-03	3.4402e-03	3.3212e-03	3.4063e-03
g_3: $\langle \lVert x-x^* \rVert \rangle$	1.0506e-01	1.4458e-02	8.7432e-03	4.9848e-03	3.9178e-03	3.7848e-03
g_4: $\langle \lVert x-x^* \rVert \rangle$	1.1399e-01	1.1675e-02	5.8082e-03	3.5469e-03	3.3044e-03	3.2542e-03

Table legends: k – neighborhood size; e – percentage of leaders; x – individual strategy obtained from simulation; $\langle \lVert x-x^* \rVert \rangle$ -- average difference between individual strategy x and group equilibrium strategy x*

SR3-Table-2: Convergence of distancing strategies by following the leaders

g_1: $\delta_i = 0.00$, g_2: $\delta_i = 0.25$, g_3: $\delta_i = 0.75$, g_4: $\delta_i = 1.00$; network randomness: b = 0.30; perturbation: $\rho = 0.20$

<table>
<tr><td>e = 0.10 \ k =</td><td>1</td><td>2</td><td>3</td><td>4</td><td>5</td><td>6</td></tr>
<tr><td>g_1: <||x-x*||></td><td>1.0290e-01</td><td>5.0156e-03</td><td>1.1352e-03</td><td>2.7322e-04</td><td>1.6418e-04</td><td>1.4374e-04</td></tr>
<tr><td>g_2: <||x-x*||></td><td>9.5124e-02</td><td>5.0103e-03</td><td>1.4289e-03</td><td>4.2806e-04</td><td>2.8337e-04</td><td>3.0476e-04</td></tr>
<tr><td>g_3: <||x-x*||></td><td>1.0316e-01</td><td>8.5974e-03</td><td>2.9661e-03</td><td>5.3369e-04</td><td>2.0126e-04</td><td>1.1416e-04</td></tr>
<tr><td>g_4: <||x-x*||></td><td>1.1069e-01</td><td>6.3729e-03</td><td>2.4610e-03</td><td>1.3038e-03</td><td>9.0332e-04</td><td>7.1867e-04</td></tr>
</table>

<table>
<tr><td>e = 0.20 \ k =</td><td>1</td><td>2</td><td>3</td><td>4</td><td>5</td><td>6</td></tr>
<tr><td>g_1: <||x-x*||></td><td>1.0527e-01</td><td>5.3157e-03</td><td>1.4115e-03</td><td>2.8662e-04</td><td>1.7882e-04</td><td>1.6491e-04</td></tr>
<tr><td>g_2: <||x-x*||></td><td>9.6341e-02</td><td>5.2177e-03</td><td>1.5554e-03</td><td>4.7324e-04</td><td>3.3156e-04</td><td>3.7131e-04</td></tr>
<tr><td>g_3: <||x-x*||></td><td>1.0484e-01</td><td>8.8606e-03</td><td>3.1150e-03</td><td>6.2236e-04</td><td>2.2149e-04</td><td>1.4473e-04</td></tr>
<tr><td>g_4: <||x-x*||></td><td>1.1286e-01</td><td>6.8585e-03</td><td>2.6128e-03</td><td>1.3928e-03</td><td>9.7815e-04</td><td>7.9031e-04</td></tr>
</table>

<table>
<tr><td>e = 0.30 \ k =</td><td>1</td><td>2</td><td>3</td><td>4</td><td>5</td><td>6</td></tr>
<tr><td>g_1: <||x-x*||></td><td>1.0697e-01</td><td>5.2959e-03</td><td>1.4053e-03</td><td>3.2107e-04</td><td>2.0438e-04</td><td>1.7429e-04</td></tr>
<tr><td>g_2: <||x-x*||></td><td>9.6208e-02</td><td>5.1737e-03</td><td>1.6113e-03</td><td>5.3018e-04</td><td>3.7392e-04</td><td>3.7349e-04</td></tr>
<tr><td>g_3: <||x-x*||></td><td>1.0436e-01</td><td>8.8107e-03</td><td>3.3167e-03</td><td>7.9975e-04</td><td>3.3439e-04</td><td>1.9636e-04</td></tr>
<tr><td>g_4: <||x-x*||></td><td>1.1279e-01</td><td>6.8770e-03</td><td>2.6331e-03</td><td>1.4495e-03</td><td>9.9627e-04</td><td>8.1506e-04</td></tr>
</table>

<table>
<tr><td>e = 0.40 \ k =</td><td>1</td><td>2</td><td>3</td><td>4</td><td>5</td><td>6</td></tr>
<tr><td>g_1: <||x-x*||></td><td>1.0661e-01</td><td>5.1010e-03</td><td>1.3578e-03</td><td>3.3491e-04</td><td>2.1811e-04</td><td>2.1162e-04</td></tr>
<tr><td>g_2: <||x-x*||></td><td>9.6984e-02</td><td>5.1143e-03</td><td>1.6073e-03</td><td>6.0607e-04</td><td>4.2467e-04</td><td>4.0139e-04</td></tr>
<tr><td>g_3: <||x-x*||></td><td>1.0473e-01</td><td>9.2232e-03</td><td>3.4776e-03</td><td>8.4719e-04</td><td>3.8942e-04</td><td>2.7492e-04</td></tr>
<tr><td>g_4: <||x-x*||></td><td>1.1289e-01</td><td>7.2024e-03</td><td>2.6792e-03</td><td>1.5047e-03</td><td>1.0069e-03</td><td>8.2175e-04</td></tr>
</table>

<table>
<tr><td>e = 0.50 \ k =</td><td>1</td><td>2</td><td>3</td><td>4</td><td>5</td><td>6</td></tr>
<tr><td>g_1: <||x-x*||></td><td>1.0667e-01</td><td>5.2341e-03</td><td>1.4287e-03</td><td>3.5962e-04</td><td>2.2961e-04</td><td>2.1686e-04</td></tr>
<tr><td>g_2: <||x-x*||></td><td>9.6655e-02</td><td>5.0973e-03</td><td>1.5877e-03</td><td>5.7760e-04</td><td>4.0984e-04</td><td>4.3852e-04</td></tr>
<tr><td>g_3: <||x-x*||></td><td>1.0394e-01</td><td>9.3438e-03</td><td>3.6229e-03</td><td>9.1973e-04</td><td>4.2352e-04</td><td>2.9077e-04</td></tr>
<tr><td>g_4: <||x-x*||></td><td>1.1163e-01</td><td>7.1687e-03</td><td>2.6836e-03</td><td>1.5319e-03</td><td>1.0284e-03</td><td>8.7435e-04</td></tr>
</table>

Table legends: k – neighborhood size; e – percentage of leaders; x – individual strategy obtained from simulation; <||x-x*||> -- average difference between individual strategy x and group equilibrium strategy x*

SR3-Figure-1: Convergence of distancing strategies by following the leaders

g_1 – red, g_2 – magenta, g_3 – cyan, g_4 – blue, all – stars; g_1: $\delta_i = 0.00$; g_2: $\delta_i = 0.25$; g_3: $\delta_i = 0.75$; g_4: $\delta_i = 1.00$; randomness of network: $b = 0.30$; leaders: $e = 0.30$; neighborhood size: $k = 1, 2$

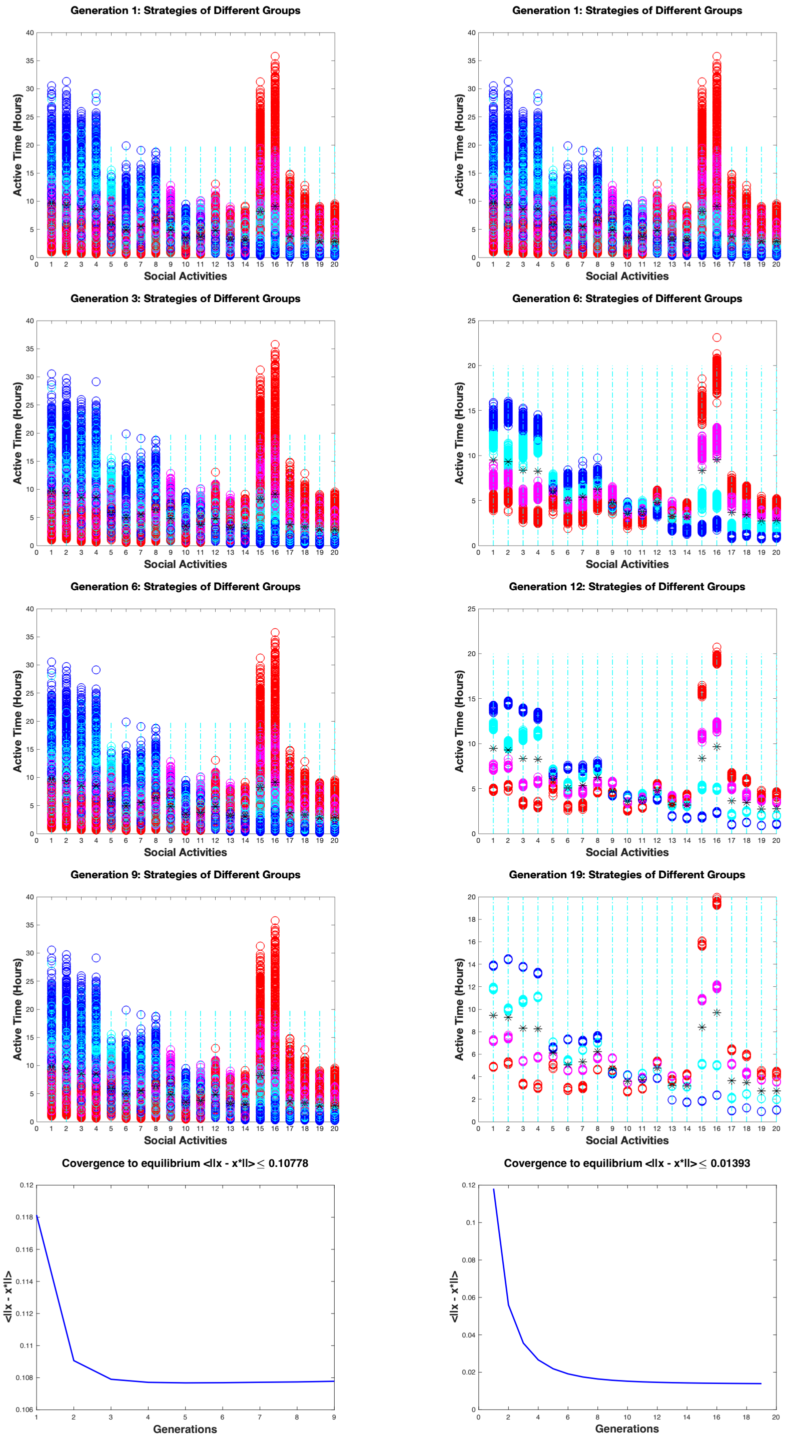

SR3-Figure-2: Convergence of distancing strategies by following the leaders

g_1 – red, g_2 – magenta, g_3 – cyan, g_4 – blue, all – stars; g_1: $\delta_i = 0.00$; g_2: $\delta_i = 0.25$; g_3: $\delta_i = 0.75$; g_4: $\delta_i = 1.00$; randomness of network: $b = 0.30$; leaders: $e = 0.30$; neighborhood size: $k = 3, 4$

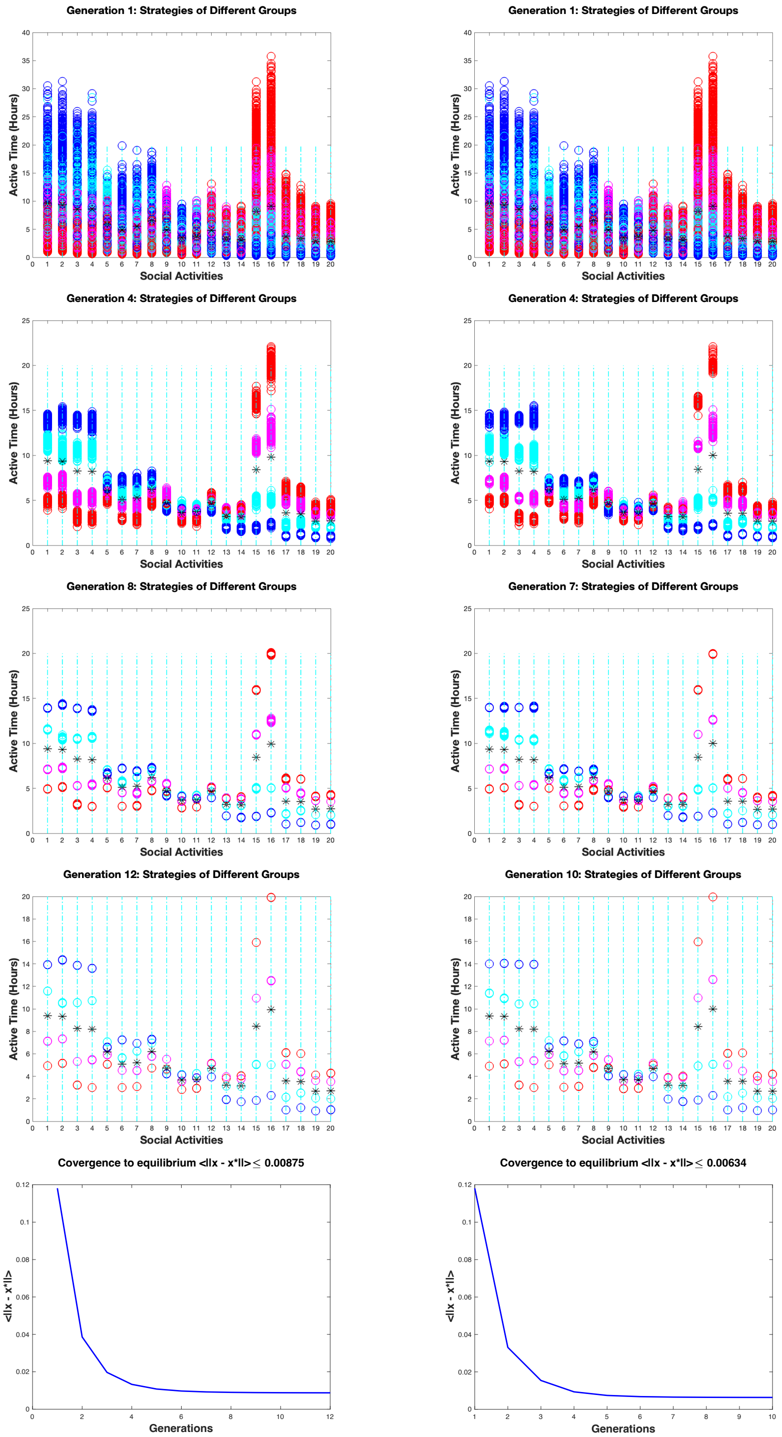

SR3-Figure-3: Convergence of distancing strategies by following the leaders

g_1 – red, g_2 – magenta, g_3 – cyan, g_4 – blue, all – stars; g_1: $\delta_i = 0.00$; g_2: $\delta_i = 0.25$; g_3: $\delta_i = 0.75$; g_4: $\delta_i = 1.00$; randomness of network: b = 0.30; leaders: e = 0.30; neighborhood size: k = 5, 6

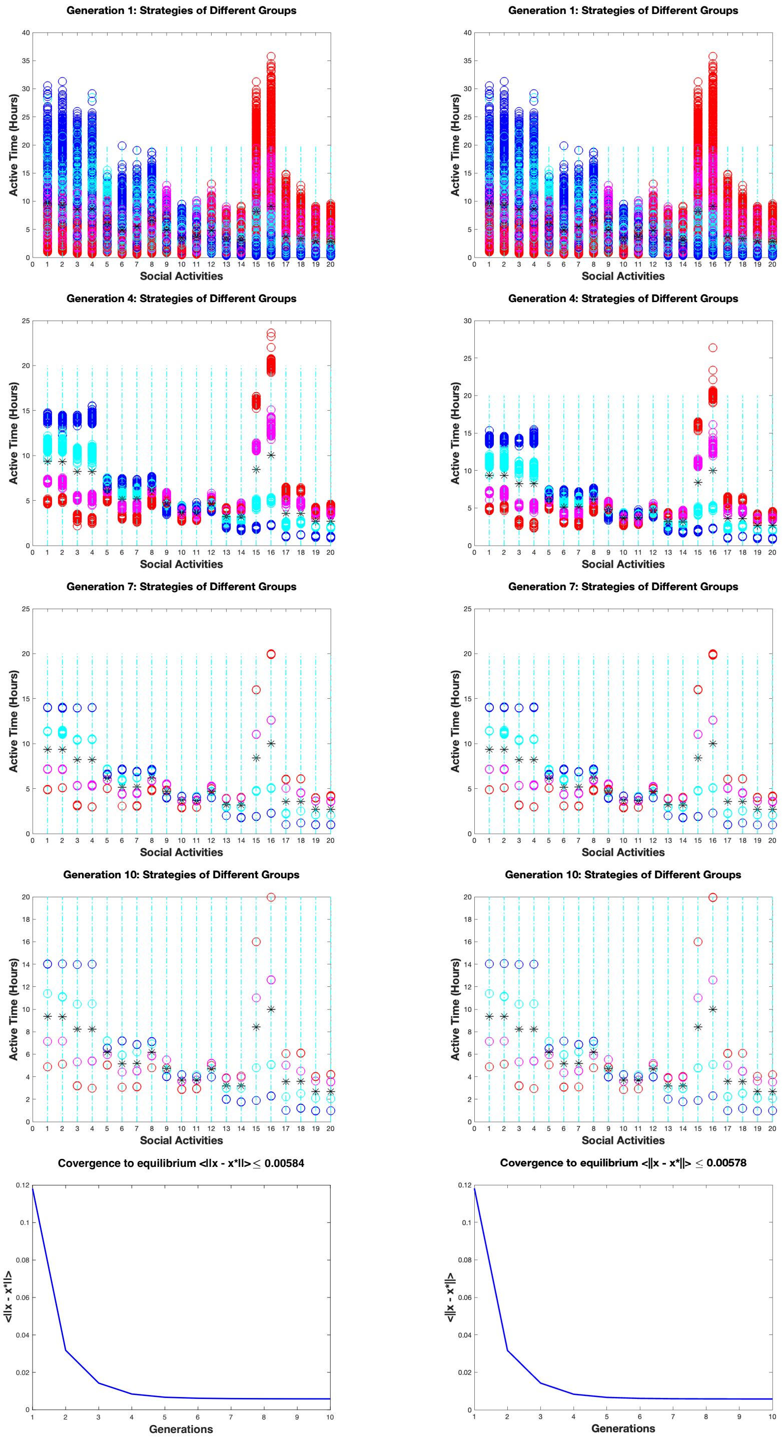

Supplement: S3 Text — (PDF) [file pone.0293489.s003.pdf]
