# Supplementary material for: Beyond six feet: The collective behavior of social distancing
Source: PLoS One. 2024 Sep 13;19(9):e0293489. doi: 10.1371/journal.pone.0293489 (PMC11398703; doi:10.1371/journal.pone.0293489)
Supplement: S1 File — (PDF) [file pone.0293489.s004.pdf]

```
% READ FIRST:
%
% To run the simulation, simply type the following function
%
% > SI_simulation_code_1
%
% There are places for program pauses. Type any key to continue.
% The simulation repeats for five times.
%
% Default parameters:
%
% severity parameter: delta = 0.50*ones(n,1)
%
% parameters for logistic function:
%
% kappa = 10*ones(n,1), theta = 0.5*ones(n,1)
%
% network: m = 2000, K = 6, b = 0.30
% neighborhood size: k = 3
%
% initial perturbation: rho = 0.20
%
% To change parameters:
%
% For severity parameter, modify:
% delta = 0.50*ones(n,1);
%
% For logistic function, modify:
% kappa = 10*ones(n,1), theta = 0.5*ones(n,1)
%
% For network, modify:
% m = 2000; K = 6; b = 0.3;
%
% For neighborhood size, modify:
% neighborhood_size = 3;
%
% For initial perturbation, modify:
% rho = 0.20;
%

function ...
[ret_info,ind_eq_compare_average,pop_eq_compare_average] ...
= SI__simulation__code_1 ()

%
%    Simulation of social distancing in small-world social networks
%
%    m individuals to participate in n activities
%
%    The population forms a small-world network.
%
%    alpha -- contact factors, n x 1
%    beta -- impact factors, n x 1
%    delta -- parameters balancing contacts and impacts, n x 1
%
%    social_network -- The adjacency matrix of the small-world
%    network, m x m
%    social_neighbors -- The index matrix of the neighbors of
%    each individual, m x m
%
%    social_neighbors(i,j) =
%    social_neighbors(j,i) = 1/k if i and j are neighbors of
%          distance k, 0 if not.
%
%    computed_eq_strategy --
%    equilibrium strategy computed based on the general model,
%    n x 1
%
```

```
%    lambda -- distancing risk at equilibrium;
%    lambda_contact -- risk of close contacts;
%    lambda_impact -- risk of negative impacts
%
%    ind_eq_strategy -- simulated individual strategies,
%    n x m
%    pop_eq_strategy -- simulated neighborhood strategies,
%    n x m
%
%    ind_eq_compare --
%    simulated individual strategy compared with computed
%    equilibrium strategy
%
%    pop_eq_compare --
%    simulated neighborhood strategy compared with computed
%    equilibrium strategy
%
%    The simulation is run for N times, with average comparison
%    results, ind_eq_compare_average, pop_eq_compare_average
%    as output.
%
%    Zhijun Wu, 04/01/2024, Math Dept, Iowa State University
%

n = 20; rng ('default');

%    Set up values for contact factors:

kappa = 10*ones(n,1); theta = 0.5*ones(n,1);

%    Set up values for contact factors

alpha = [14 14 14 14 7 7 7 7 4 4 4 4 2 2 2 2 1 1 1 1]';
alpha = 1 + exp(-kappa .* (alpha / sum(alpha) - theta));
lambda = 1 / min(alpha); alpha = lambda * alpha;

%    Set up values for impact factors

beta = [5 5 3 3 5 3 3 5 5 3 3 5 4 4 16 20 6 6 4 4]';
beta = 1 + exp(-kappa .* (beta / sum(beta) - theta));
beta = lambda * beta;

%    Set up values for severity parameters:

delta = 0.50*ones(n,1);

%    Compute equilibrium strategy

[computed_eq_strategy,lambda,lambda_contact,lambda_impact] ...
= compute_eq_strategy(alpha,beta,delta,kappa,theta);

format short;

disp(' '); disp('Computed equilibrium strategy: ');
disp(' '); disp(112*computed_eq_strategy);
disp(' '); disp('Contacts and impacts at equilibrium: ')
disp(' '); disp(['lambda: ',num2str(2000*lambda),'; ',...
'lambda_contact: ',num2str(2000*lambda_contact),'; ',...
'lambda_impact: ',num2str(2000*lambda_impact)]);

%    Generate the small-world social network

m = 2000; K = 6; b = 0.30;
social_network = small_world_network(m,K,b);

%    Determine neighborhood for every individual:

neighborhood_size = 3;
```

```
social_neighbors = network_neighbors(social_network,...
neighborhood_size);

%    Perturb computed equilibrium strategy:

rho = 0.20; perturbed_eq_strategy = zeros(n,1);
for i = 1 : n
    perturbed_eq_strategy(i,1) = ...
    computed_eq_strategy(i,1) * (1 + 2 * rho * (0.5 - rand));
end
perturbed_eq_strategy = perturbed_eq_strategy ...
/ sum(perturbed_eq_strategy);

%    Repeat simulation with different initial strategies

N = 5; ind_eq_compare_average = 0; pop_eq_compare_average = 0;

for i = 1 : N

    % Random initial frequencies

    initial_eq_strategy(1:n,1:m) = rand(n,m);

    % Normalize the frequencies

    for l = 1 : m
        initial_eq_strategy(1:n,l) = ...
        perturbed_eq_strategy + 1.6 * perturbed_eq_strategy ...
        .* (0.5 - initial_eq_strategy(1:n,l));
        initial_eq_strategy(1:n,l) = initial_eq_strategy(1:n,l) ...
        / sum(initial_eq_strategy(1:n,l));
    end

    % Start simulation, to reach equilibrium strategies

    [ind_eq_strategy,pop_eq_strategy] ...
    = simulation_1(initial_eq_strategy,computed_eq_strategy,...
    social_neighbors,alpha,beta,delta,kappa,theta);

    ind_eq_compare = sum (sqrt (sum ((ind_eq_strategy - ...
    computed_eq_strategy * ones(1,m)).^2))) / m;
    ind_eq_compare_average = ...
    ind_eq_compare_average + ind_eq_compare;

    pop_eq_compare = sum (sqrt (sum ((pop_eq_strategy - ...
    computed_eq_strategy * ones(1,m)).^2))) / m;
    pop_eq_compare_average = pop_eq_compare_average + ...
    pop_eq_compare;

end

ind_eq_compare_average = ind_eq_compare_average / N;
pop_eq_compare_average = pop_eq_compare_average / N;

format shorte;

disp(' ');
disp('Individual strategy, simulated vs computed: ')
disp(' ');
disp(['ind_eq_compare_average = ',...
num2str(ind_eq_compare_average)]);
disp(' ');
disp('Population strategy, simulated vs computed: ')
disp(' ');
disp(['pop_eq_compare_average = ',...
num2str(pop_eq_compare_average)]);

ret_info = 1;
```

```
end

%%%%%%%%%%%%%%%%%%%%%%%%%%%%%%%%%%%%%%%%%%%%%%%%%%%%%%%%%%%%%%%%


function ...
[computed_eq_strategy,lambda,lambda_contact,lambda_impact] ...
= compute_eq_strategy (alpha,beta,delta,kappa,theta)

%   Computing equilibrium strategy
%
%   Compute the equilibrium strategy based on the general ...
%   game model
%
%   Input: Parameters: alpha, beta, delta, kappa, theta ...
%   for functions on distancing risks
%
%   Output: Equilibrium strategy: computed_eq_strategy
%   Distancing risks: lambda, lambda_contact, lambda_impact

omega = delta .* alpha + (1 - delta) .* beta;

lb = 0.00001; ub = min(omega)-0.00001; lambda0 = 0.001;

options = optimoptions('lsqnonlin','Algorithm',...
'levenberg-marquardt','SpecifyObjectiveGradient',true);

lambda = lsqnonlin(@(lambda) lambda_equation ...
(lambda,omega,kappa,theta),lambda0,lb,ub,options);

computed_eq_strategy = theta + log(lambda ./ ...
(omega - lambda)) ./ kappa;

lambda_contact = computed_eq_strategy' * (alpha ./ ...
(1 + exp(-kappa .* (computed_eq_strategy - theta))));
lambda_impact = computed_eq_strategy' * (beta ./ ...
(1 + exp(-kappa .* (computed_eq_strategy - theta))));

end

%%%%%%%%%%%%%%%%%%%%%%%%%%%%%%%%%%%%%%%%%%%%%%%%

function [F,J]...
= lambda_equation(lambda,omega,kappa,theta)

F = sum((log(lambda) - log(omega - lambda)) ./ ...
kappa) + sum(theta) - 1;
J = sum((1 ./ lambda + 1 ./ (omega - lambda)) ...
./ kappa);

end


%%%%%%%%%%%%%%%%%%%%%%%%%%%%%%%%%%%%%%%%%%%%%%%%%%%%%%%%%%%%%%%%%%

function network = small_world_network (N,K,b)

%
%   Generation of Small-World Population Network
%
%   N -- population size
%   K -- degree of connection, even number
%   b -- randomness parameter, [0,1]
%
%   network -- adjacency matrix of population network
%
%   Zhijun Wu, 04/01/2024, Math Dept, Iowa State University
```

```
%

network = zeros(N);

for i = 0 : N-1
    k = 1;
    while (k <= K/2)
        j = mod(i+k,N);
        network(i+1,j+1) = 1;
        network(j+1,i+1) = 1;
        k = k +1;
    end
    k = 1;
    while (k <= K/2)
        j = mod(i-k,N);
        network(i+1,j+1) = 1;
        network(j+1,i+1) = 1;
        k = k + 1;
    end
end

for i = 0 : N-1
    k = 1;
    while (k <= K/2)
        j = mod(i+k,N);
        if (network(i+1,j+1) == 1)
           l = floor(N*rand);
           while (l == i || network(i+1,l+1) == 1)
              l = floor(N*rand);
           end
           if (rand <= b)
              network(i+1,l+1) = 1;
              network(l+1,i+1) = 1;
              network(i+1,j+1) = 0;
              network(j+1,i+1) = 0;
           end
        end
        k = k +1;
    end
end

end

%%%%%%%%%%%%%%%%%%%%%%%%%%%%%%%%%%%%%%%%%%%%%%%%%%%%%%%%%%%%%%%%%%

function [neighbors,average_neighbors] ...
= network_neighbors (network,neighborhood_size)

%
%   Generate neighborhood index
%
%   neighborhood_size -- neighborhood size
%
%   network -- adjacency matrix of population network
%
%   neighbors -- matrix to represent neighborhood of
%   neighborhood_size:
%   neighbors(i,j) = 1 if there is a path of length <=
%.  neighborhood_size connecting i and j.
%
%   Zhijun Wu, 04/01/2024, Math Dept, Iowa State University
%

N = size(network,1);

neighbors_k = zeros(N,N,neighborhood_size);
neighbors_k(1:N,1:N,1) = network;
neighbors = neighbors_k(1:N,1:N,1) + eye(N);
```

```
% The (i,j)-element of the k-th power of adj matrix A
% gives the number of walks from i to j of length k.

for k = 2 : neighborhood_size
    neighbors_k(1:N,1:N,k) = neighbors_k(1:N,1:N,k-1) * network;
    for i = 1 : N
        for j = 1 : N
            if (neighbors_k(i,j,k) > 0)
               if (neighbors(i,j) == 0)
                  neighbors(i,j) = 1/k;
    % higher weights assigned to closer neighbors
               end
            end
        end
    end
end

average_neighbors = nnz(neighbors) / N;

end

%%%%%%%%%%%%%%%%%%%%%%%%%%%%%%%%%%%%%%%%%%%%%%%%%%%%%%%%%%%%%%%%%%
% READ FIRST:
%
% To silence displays, comment out:
% display_group_strategy(...);
%

function ...
[ind_eq_strategy,pop_eq_strategy] = simulation_1 ...
(initial_eq_strategy,computed_eq_strategy,social_neighbors,...
alpha,beta,delta,kappa,theta)

%
%   Simulation of social distancing in small-world
%   social networks
%
%   m individuals to visit n social activities:
%
%   alpha -- contact factors, n x 1
%   beta -- impact factors, n x 1
%   delta -- severity parameters, n x 1
%   kappa -- parameters for logistic function
%   theta -- parameters for logistic function
%
%   initial_eq__strategy
%   -- initial individual strategy, n x m
%   computed_eq_strategy
%.  -- computed equilibrium strategy, n x 1
%
%   social_neighbors -- index matrix for social neighbors
%
%   Zhijun Wu, 04/01/2024, Math Dept, Iowa State University
%

n = size(initial_eq_strategy,1);
m = size(initial_eq_strategy,2);

ind_eq_strategy = initial_eq_strategy;
pop_eq_strategy = initial_eq_strategy;

for l = 1 : m
    pop_eq_strategy(1:n,l) = ind_eq_strategy(1:n,1:m) ...
    * social_neighbors(1:m,l) / sum(social_neighbors(1:m,l));
end

display_ind_strategy(ind_eq_strategy,1);
```

```
display_pop_strategy(pop_eq_strategy,1);

% Initial and maximum # iterations

k = 1; K = 200;

ind_eq_average = sum(ind_eq_strategy,2) / m;
ind_eq_compare = zeros(K,1);
ind_eq_compare(k) = sum ( sqrt( sum((ind_eq_strategy - ...
computed_eq_strategy * ones(1,m)).^2))) / m;

ind_eq_increase = 1; ind_eq_change = 1;

ind_eq_average_5 = zeros(n,5);
ind_eq_average_5(1:n,5) = ind_eq_average;

while (ind_eq_compare(k) > 1.0e-4 && ind_eq_change > ...
1.0e-5 && ind_eq_increase >= 0 && k <= K)

   for j = 1 : m

       x = ind_eq_strategy(1:n,j);
       w = social_neighbors(1:m,j); w = w / sum(w);
       y = ind_eq_strategy(1:n,1:m) * w;

       ind_eq_strategy(1:n,j) = ...
       update_strategy (x,y,alpha,beta,delta,kappa,theta);

   end

   k = k + 1;

   for l = 1 : m
       pop_eq_strategy(1:n,l) = ind_eq_strategy(1:n,1:m) ...
       * social_neighbors(1:m,l) / sum(social_neighbors(1:m,l));
   end

   ind_eq_average = sum(ind_eq_strategy,2) / m;
   for l = 1 : 4
       ind_eq_average_5(1:n,l) = ind_eq_average_5(1:n,l+1);
   end
   ind_eq_average_5(1:n,5) = ind_eq_average;

   mu = sum(ind_eq_average_5,2) / 5;
   dev = ind_eq_average_5 - mu * ones(1,5);
   ind_eq_change = sum (sqrt (sum (dev.^2) / n)) / 5;

   ind_eq_compare(k) = sum (sqrt (sum ((ind_eq_strategy - ...
   computed_eq_strategy * ones(1,m)).^2))) / m;

   if k > 5
      ind_eq_increase = 0;
      for l = 1 : 5
          ind_eq_increase = ind_eq_increase + ...
          ind_eq_compare(k-l) - ind_eq_compare(k-l+1);
      end
      ind_eq_increase = ind_eq_increase / 5;
   end

   % display strategies once every l iterations:

   l = 1;
   if (mod(k,l) == 0)
      display_ind_strategy(ind_eq_strategy,k);
      display_pop_strategy(pop_eq_strategy,k);
   end

end
```

```
   plot_eq_compare(ind_eq_compare,k);

end

%%%%%%%%%%%%%%%%%%%%%%%%%%%%%%%%%%%%%%

function ind_strategy_out ...
= update_strategy (ind_strategy_in,pop_strategy_in,...
alpha,beta,delta,kappa,theta)

%
%   Update individual distancing strategy
%
%   ind_strategy_in -- current individual strategy, n x 1
%   pop_strategy_in -- current population strategy, n x 1
%
%   alpha -- contact factors, n x 1
%   beta -- impact factors, n x 1
%   delta -- severity parameters, n x 1
%
%   ind_strategy_out -- updated individual strategy, n x 1
%
%
%   Zhijun Wu, 04/01/2024, Math Dept, Iowa State University
%

n = size(ind_strategy_in,1);

x = ind_strategy_in;
y = pop_strategy_in;

% pot_risk -- potential distancing risk;
% pop_risk -- distancing risk of the population;

omega = delta .* alpha + (1 - delta) .* beta;
pot_risk = omega ./ (1 + exp(-kappa .* (y - theta)));
pop_risk = y' * pot_risk;

   for i = 1 : n

   % strategy i has lower contact, increase its frequency

       if (pop_risk > pot_risk(i))
          if (x(i) < y(i))
             x(i) = x(i) + 0.9 * (y(i) - x(i));
             % * (pop_risk - pot_risk(i)) / pop_risk;
          else
             x(i) = x(i) + 0.1 * min(x(i)-y(i),1.0-x(i));
             % * (pop_risk - pot_risk(i)) / pop_risk;
          end
       end

       % strategy i has higher contact, reduce its frequency:

       if (pop_risk < pot_risk(i))
          if (x(i) > y(i))
             x(i) = x(i) - 0.9 * (x(i) - y(i));
             % * (pot_risk(i) - pop_risk) / pot_risk(i);
          else
             x(i) = x(i) - 0.1 * min(y(i)-x(i),x(i)-0.0);
             % * (pot_risk(i) - pop_risk) / pot_risk(i);
          end
       end

       % pot_risk at i is close to pop_risk,
       % adjust frequency to y(i):
```

```
        if (abs(pop_risk - pot_risk(i)) < 0.01)
            if (x(i) > y(i))
                x(i) = x(i) - 0.5 * (x(i) - y(i));
            end
            if (x(i) < y(i))
                x(i) = x(i) + 0.5 * (y(i) - x(i));
            end
        end


    end

ind_strategy_out = x / sum(x);

end

%%%%%%%%%%%%%%%%%%%%%%%%%%%%%%%%%%%%%%%%%%%%%%

function display_ind_strategy (ind_strategy,g)

acts_0 = ...
[0 1 2 3 4 5 6 7 8 9 10 11 12 13 14 15 16 17 18 19 20];

[n,m] = size(ind_strategy);

ind_average = sum (ind_strategy,2) / m;
ind_deviation = sqrt (sum ((ind_strategy - ind_average ...
* ones(1,m)).^2, 2) / m);
ind_deviation = sum (ind_deviation ./ ind_average) / n;

plot(ind_strategy*112,'ob','MarkerSize',12,'LineWidth',1);
hold on;

xticks(acts_0);

title(['Generation ',num2str(g),': ','STD Individual = ',...
num2str(ind_deviation,'%0.5f\n')],'FontSize',16,...
'FontWeight','Bold');
xlabel('Social Activities','FontSize',16,...
'FontWeight','Bold');
ylabel('Active Time (Hours)','FontSize',16,...
'FontWeight','Bold');

hold off;
pause;

end

%%%%%%%%%%%%%%%%%%%%%%%%%%%%%%%%%%%%%%%%%%%%%%

function display_pop_strategy (pop_strategy,g)

acts_0 = ...
[0 1 2 3 4 5 6 7 8 9 10 11 12 13 14 15 16 17 18 19 20];

[n,m] = size(pop_strategy);

pop_average = sum (pop_strategy,2) / m;
pop_deviation = sqrt (sum ((pop_strategy - pop_average * ...
ones(1,m)).^2, 2) / m);
pop_deviation = sum (pop_deviation ./ pop_average) / n;

plot(pop_strategy*112,'ob','MarkerSize',12,'LineWidth',1);
hold on;

xticks(acts_0);

title(['Generation ',num2str(g),': ','STD Population = ', ...
```

```
num2str(pop_deviation,'%0.5f\n')],'FontSize',16,...
'FontWeight','Bold');
xlabel('Social Activities','FontSize',16,...
'FontWeight','Bold');
ylabel('Active Time (Hours)','FontSize',16,...
'FontWeight','Bold');

hold off;
pause;

end

%%%%%%%%%%%%%%%%%%%%%%%%%%%%%%%%%%%%%%%%%%%%%%%%%%%

function plot_eq_compare (strategy_eq_compare,k)

plot(strategy_eq_compare(1:k),'-b','LineWidth',2);
hold on;

title(['Covergence to Equilibrium <||x - x*||> \leq ',...
num2str(strategy_eq_compare(k),'%0.5f\n')],...
' ','FontSize',16);
xlabel('Generations','FontSize',16,'FontWeight','bold');
ylabel('<||x - x*||>','FontSize',16,'FontWeight','bold');

ax = gca;
ax.XTick = unique(round(ax.XTick));

hold off;
pause;

end
```
