# Supplementary material for: Beyond six feet: The collective behavior of social distancing
Source: PLoS One. 2024 Sep 13;19(9):e0293489. doi: 10.1371/journal.pone.0293489 (PMC11398703; doi:10.1371/journal.pone.0293489)
Supplement: S2 File — (PDF) [file pone.0293489.s005.pdf]

```
% READ FIRST:
%
% To run the simulation, simply type the following function
%
% > SI_simulation_code_2
%
% There are places for program pauses.
% Type any key to continue.
% The simulation repeats for five times.
%
% Default parameters:
%
% severity parameters:
% delta(:,1) = 0.00, delta(:,2) = 0.25,
% delta(:,3) = 0.75, delta(:,4) = 1.00
%
% parameters for logistic function:
% kappa = 10*ones(n,1), theta = 0.5*ones(n,1)
%
% network: m = 2000, K = 6, b = 0.30
% neighborhood size: k = 3
%
% initial perturbation: rho = 0.20
%
% To change parameters:
%
% For balancing parameter, modify:
%
% delta(1:n,1) =
% 0.00*ones(n,1); delta(1:n,2) = 0.25*ones(n,1);
% delta(1:n,3) =
% 0.75*ones(n,1); delta(1:n,4) = 1.00*ones(n,1);
%
% For logistic function, modify:
% kappa = 10*ones(n,1), theta = 0.5*ones(n,1)
%
% For network, modify:
% m = 2000; K = 6; b = 0.3;
%
% For neighborhood size, modify:
% neighborhood_size = 3;
%
% For initial perturbation, modify:
% rho = 0.20;
%

function ...
[ret_info,ind_eq_compare_average,pop_eq_compare_average] ...
= SI_simulation_code_2 ()

%
%    Simulation of social distancing
%    in multiple population groups
%
%    m individuals in M groups to participate
%    in n activities
%
%    The population is divided into M groups
%    and distributed over a small-world network.
%
%    alpha -- contact facors, n x 1
%    beta -- impact factors, n x 1
%
%    delta --
%    parameters balancing contact and impact, n x M
%    delta(1:n,l) is used for group l.
%
%    social_network -- The adjacency matrix of
```

```
%    the small-world network, m x m
%    social_neighbors -- The index matrix of the neighbors
%    of each individual, m x m
%
%    social_neighbors(i,j) =
%    social_neighbors(j,i) = 1/k if i and j are neighbors
%    of distance k, 0 if not.
%
%    pop_groups -- index matrix for population groups
%    pop_groups(j,l) =
%    1 if individual j is in group l, 0 if not.
%
%    computed_eq_strategy -- equilibrium strategy
%    computed based on general model, n x M,
%    and computed_eq_strategy(1:n,l) is for group l.
%
%    lambda -- distancing risk at equilibrium;
%    lambda_contact -- risk of close contacts;
%    lambda_impact -- risk of negative impacts
%
%    ind_eq_strategy --
%    simulated individual strategies, n x m
%    pop_eq_strategy --
%    simulated population strategies, n x m
%
%    ind_eq_compare --
%    simulated group strategy compared with
%    computed group equilibrium strategy
%
%    pop_eq_compare --
%    simulated population strategy compared with
%    computed population equilibrium strategy
%
%    The simulation is run for N times,
%    with average comparison results, ind_eq_compare_average,
%    pop_eq_compare_average as output.
%
%    Zhijun Wu, 04/01/2024, Math Dept, Iowa State University
%

n = 20; rng ('default');

%    Set up parameters for logistic function

kappa = 10*ones(n,1); theta = 0.5*ones(n,1);

%    Set up values for contact factors

alpha = [14 14 14 14 7 7 7 7 4 4 4 4 2 2 2 2 1 1 1 1]';
alpha = 1 + exp(-kappa .* (alpha / sum(alpha) - theta));
lambda = 1 / min(alpha); alpha = lambda * alpha;

%    Set up values for impact factors

beta = [5 5 3 3 5 3 3 5 5 3 3 5 4 4 16 20 6 6 4 4]';
beta = 1 + exp(-kappa .* (beta / sum(beta) - theta));
beta = lambda * beta;

%    Set up severity parameters:

delta(1:n,1) = 0.00*ones(n,1); delta(1:n,2) = 0.25*ones(n,1);
delta(1:n,3) = 0.75*ones(n,1); delta(1:n,4) = 1.00*ones(n,1);

%    Generate the small-world social network

m = 2000; K = 6; b = 0.30;
social_network = small_world_network(m,K,b);
```

```
%    Determine neighborhood for every individual:

neighborhood_size = 3;
social_neighbors = ...
network_neighbors(social_network,neighborhood_size);

%    Compute equilibrium strategy

M = 4;

pop_groups = zeros(m,M);
for l = 1 : M
    for j = 1 : m
        if (mod(j,M) == mod(l,M))
           pop_groups(j,l) = 1;
        end
    end
end

computed_eq_strategy = zeros(n,M);
lambda = zeros(M,1); lambda_contact = zeros(M,1);
lambda_impact = zeros(M,1);

for l = 1 : M
    [computed_eq_strategy(1:n,l),lambda(l),...
    lambda_contact(l),lambda_impact(l)] ...
    = compute_eq_strategy(alpha,beta,delta(1:n,l),kappa,theta);
end

format short;

for l = 1 : M

    disp(' ');
    disp(['Computed equilibrium strategy for Group ',...
    num2str(l),': ']);
    disp(' ');
    disp(112*computed_eq_strategy(1:n,l));
    disp(' ');
    disp(['Contacts and impacts at equilibrium for Group ',...
    num2str(l),': '])
    disp(' ');
    disp(['lambda: ',num2str(2000*lambda(l)),'; ',...
    'lambda_contact: ',num2str(2000*lambda_contact(l)),...
    '; ','lambda_impact: ',num2str(2000*lambda_impact(l))]);

end

%    Perturb computed equilibrium strategy:

rho = 0.20; perturbed_eq_strategy = zeros(n,M);
for l = 1 : M
    for i = 1 : n
        perturbed_eq_strategy(i,l) = ...
        computed_eq_strategy(i,l) * ...
        (1 + 2 * rho * (0.5 - rand));
    end
    perturbed_eq_strategy(1:n,l) = ...
    perturbed_eq_strategy(1:n,l) / ...
    sum(perturbed_eq_strategy(1:n,l));
end

%    Repeat simulation with different initial strategies

ind_eq_compare_average = zeros(M,1);
pop_eq_compare_average = 0;

computed_eq_average = sum(computed_eq_strategy,2) / M;
```

```
N = 5;

for i = 1 : N

    % Generate initial strategies

    initial_eq_strategy(1:n,1:m) = rand(n,m);

    % Normalize the frequencies

    for j = 1 : m
        for l = 1 : M
            if (pop_groups(j,l) == 1)
               k = l;
            end
        end
        initial_eq_strategy(1:n,j) = ...
        perturbed_eq_strategy(1:n,k) + 1.6 * ...
        perturbed_eq_strategy(1:n,k) .* ...
        (0.5 - initial_eq_strategy(1:n,j));
        initial_eq_strategy(1:n,j) = ...
        initial_eq_strategy(1:n,j) / ...
        sum(initial_eq_strategy(1:n,j));
    end

    % Start simulation, to reach equilibrium strategies

    ind_eq_strategy = simulation_2 ...
    (initial_eq_strategy,computed_eq_strategy,...
     social_neighbors,pop_groups,alpha,...
     beta,delta,kappa,theta);

    ind_eq_average = zeros(n,M); ind_eq_compare = zeros(M,1);

    for l = 1 : M
        ind_eq_average(1:n,l) = ind_eq_strategy(1:n,1:m) * ...
        pop_groups(1:m,l) / (m/M);
    end

    for l = 1 : M
        group_eq_strategy = zeros(n,m);
        for j = 1 : m
            if (pop_groups(j,l) == 1)
               group_eq_strategy(1:n,j) = ...
               ind_eq_strategy(1:n,j) - ...
               computed_eq_strategy(1:n,l);
            end
        end
        ind_eq_compare(l) = ...
        sum( sqrt (sum (group_eq_strategy.^2))) / (m/M);
        ind_eq_compare_average(l) = ...
        ind_eq_compare_average(l) + ind_eq_compare(l);
    end

    pop_eq_average = sum (ind_eq_strategy,2) / m;
    pop_eq_compare = sqrt (sum ((pop_eq_average - ...
    computed_eq_average).^2));
    pop_eq_compare_average = pop_eq_compare_average + ...
    pop_eq_compare;

end

for l = 1 : M

    ind_eq_compare_average(l) = ...
    ind_eq_compare_average(l) / N;
```

```
    disp(' ');
    disp(...
    ['Individual strategy, simulated vs computed, in Group ',...
    num2str(l),': '])
    disp(' ');
    disp(['ind_eq_compare_average = ',...
    num2str(ind_eq_compare_average(l))]);

end

pop_eq_compare_average = pop_eq_compare_average / N;

disp(' ');
disp(['Average individual strategy, simulated vs computed,',...
' in whole population: '])
disp(' ');
disp(['ind_eq_compare_average = ',...
num2str(pop_eq_compare_average)]);

ret_info = 1;

end

%%%%%%%%%%%%%%%%%%%%%%%%%%%%%%%%%%%%%%%%%%%%%%%%%%%%%%%%%%%%

function ...
[computed_eq_strategy,lambda,lambda_contact,lambda_impact] ...
= compute_eq_strategy (alpha,beta,delta,kappa,theta)

%   Computing equilibrium strategy
%
%   Compute the equilibrium strategy based
%   on general game model
%
%   Input: Parameters: alpha, beta, delta, kappa,
%   theta for functions on distancing risks
%
%   Output: Equilibrium strategy: computed_eq_strategy
%   Distancing risks: lambda, lambda_contact, lambda_impact

omega = delta .* alpha + (1 - delta) .* beta;

lb = 0.000001; ub = min(omega)-0.000001; lambda0 = 0.0001;

options = optimoptions('lsqnonlin','Algorithm',...
'levenberg-marquardt','SpecifyObjectiveGradient',true);

lambda = lsqnonlin(@(lambda) ...
lambda_equation(lambda,omega,kappa,theta),...
lambda0,lb,ub,options);

computed_eq_strategy = theta + log(lambda ./ ...
(omega - lambda)) ./ kappa;

lambda_contact = computed_eq_strategy' * ...
(alpha ./ (1 + exp(-kappa .* ...
(computed_eq_strategy - theta))));
lambda_impact = computed_eq_strategy' * ...
(beta ./ (1 + exp(-kappa .* ...
(computed_eq_strategy - theta))));

end

%%%%%%%%%%%%%%%%%%%%%%%%%%%%%%%%%%%%%%%%%%%%%%%%

function [F,J] ...
= lambda_equation(lambda,omega,kappa,theta)
```

```
F = sum((log(lambda) - log(omega - lambda)) ...
./ kappa) + sum(theta) - 1;
J = sum((1 ./ lambda + 1 ./ (omega - lambda)) ./ kappa);

end

%%%%%%%%%%%%%%%%%%%%%%%%%%%%%%%%%%%%%%%%%%%%%%

function network = small_world_network (N,K,b)

%
%   Generation of Small-World Population Network
%
%   N -- population size
%   K -- degree of connection, even number
%   b -- randomness parameter, [0,1]
%
%   network -- adjacency matrix of population network
%
%   Zhijun Wu, 04/01/2024, Math Dept, Iowa State University
%

network = zeros(N);

for i = 0 : N-1
    k = 1;
    while (k <= K/2)
        j = mod(i+k,N);
        network(i+1,j+1) = 1;
        network(j+1,i+1) = 1;
        k = k +1;
    end
    k = 1;
    while (k <= K/2)
        j = mod(i-k,N);
        network(i+1,j+1) = 1;
        network(j+1,i+1) = 1;
        k = k + 1;
    end
end

for i = 0 : N-1
    k = 1;
    while (k <= K/2)
        j = mod(i+k,N);
        if (network(i+1,j+1) == 1)
           l = floor(N*rand);
           while (l == i || network(i+1,l+1) == 1)
              l = floor(N*rand);
           end
           if (rand <= b)
              network(i+1,l+1) = 1;
              network(l+1,i+1) = 1;
              network(i+1,j+1) = 0;
              network(j+1,i+1) = 0;
           end
        end
        k = k +1;
    end
end

end

%%%%%%%%%%%%%%%%%%%%%%%%%%%%%%%%%%%%%%%%%%%%%%%%%%%%%%%%%

function neighbors ...
= network_neighbors (network,neighborhood_size)
```

```
%
%   Generate neighborhood index
%
%   neighborhood_size -- neighborhood size
%
%   network -- adjacency matrix of population network
%
%   neighbors --
%   matrix to represent neighborhood of neighborhood_size:
%   neighbors(i,j) = 1 if there is a path of length
%   <= neighborhood_size connecting i and j.
%
%   Zhijun Wu, 04/01/2024, Math Dept, Iowa State University
%

N = size(network,1);

neighbors = zeros(N);

neighbors_k = zeros(N,N,neighborhood_size);
neighbors_k(1:N,1:N,1) = network;
neighbors = neighbors_k(1:N,1:N,1) + eye(N);

for k = 2 : neighborhood_size
    neighbors_k(1:N,1:N,k) = ...
    neighbors_k(1:N,1:N,k-1) * network;
    for i = 1 : N
        for j = 1 : N
            if (neighbors_k(i,j,k) > 0)
               if (neighbors(i,j) == 0)
                  neighbors(i,j) = 1/k;
               end
            end
        end
    end
end

end


%%%%%%%%%%%%%%%%%%%%%%%%%%%%%%%%%%%%%%%%%%%%%%%%%%%%%%%%%%%%%
% READ FIRST:
%
% To silence displays, comment out:
% display_group_strategy(...);
%

function ind_eq_strategy = simulation_2 ...
(initial_eq_strategy,computed_eq_strategy,social_neighbors,...
pop_groups,alpha,beta,delta,kappa,theta)

%
%   Simulation of social distancing
%   in multiple population groups
%
%   m individuals in M population groups to
%   visit n social activities:
%
%   alpha -- contact factors, n x 1
%   beta -- impact factors, n x 1
%   delta -- balancing parameters, n x 1
%
%   initial_eq_strategy
%   -- initial individual strategy, n x m
%   computed_eq_strategy
%   -- computed equilibrium strategy, n x M
%
%   social_neighbors
```

```
%   -- index matrix for social neighbors
%
%   Zhijun Wu, 05/01/2023, Math Dept, Iowa State University
%

[n,m] = size(initial_eq_strategy);
M = size(pop_groups,2);

ind_eq_strategy = initial_eq_strategy;

display_group_strategy(ind_eq_strategy,pop_groups,1)

k = 1; K = 200;

ind_eq_compare = zeros(K,1);

for l = 1 : M
    group_eq_strategy = zeros(n,m);
    for j = 1 : m
        if (pop_groups(j,l) == 1)
           group_eq_strategy(1:n,j) = ...
           ind_eq_strategy(1:n,j) - ...
           computed_eq_strategy(1:n,l);
        end
    end
    ind_eq_compare(k) = ind_eq_compare(k) + ...
    sum (sqrt (sum (group_eq_strategy.^2))) / (m/M);
end

ind_eq_compare(k) = ind_eq_compare(k) / M;

ind_eq_average = sum(ind_eq_strategy,2) / m;

ind_eq_average_5 = zeros(n,5);
ind_eq_average_5(1:n,5) = ind_eq_average;

ind_eq_increase = 1; ind_eq_change = 1;

while (ind_eq_compare(k) > 1.0e-4 && ...
      ind_eq_change > 1.0e-5 && ind_eq_increase ...
      >= 0 && k <= K)

   for j = 1 : m

       x = ind_eq_strategy(1:n,j);

       weights = ones(M,1);
       for l = 1 : M
           group_neighbors = social_neighbors(1:m,j) ...
           .* pop_groups(1:m,l);
           w = group_neighbors / sum (group_neighbors);
           y(1:n,l) = ind_eq_strategy(1:n,1:m) * w;
           weights(l) = sum(group_neighbors) / ...
           sum(social_neighbors(1:m,j));
       end

       for l = 1 : M
           if (pop_groups(j,l) == 1)
              group = l;
           end
       end
       ind_eq_strategy(1:n,j) = ...
       update_strategy ...
       (x,y,weights,alpha,beta,delta,kappa,theta,group);

   end

   k = k + 1;
```

```
   for l = 1 : M
       group_eq_strategy = zeros(n,m);
       for j = 1 : m
           if (pop_groups(j,l) == 1)
              group_eq_strategy(1:n,j) = ...
              ind_eq_strategy(1:n,j) - ...
              computed_eq_strategy(1:n,l);
           end
       end
       ind_eq_compare(k) = ind_eq_compare(k) + ...
       sum (sqrt (sum (group_eq_strategy.^2))) ...
       / (m/M);
   end

   ind_eq_compare(k) = ind_eq_compare(k) / M;

   ind_eq_average = sum(ind_eq_strategy,2) / m;

   for l = 1 : 4
       ind_eq_average_5(1:n,l) = ...
       ind_eq_average_5(1:n,l+1);
   end

   ind_eq_average_5(1:n,5) = ind_eq_average;
   mu = sum(ind_eq_average_5,2) / 5;
   dev = ind_eq_average_5 - mu * ones(1,5);
   ind_eq_change = sum (sqrt (sum (dev.^2) / n)) / 5;

   if k > 5
      ind_eq_increase = 0;
      for l = 1 : 5
          ind_eq_increase = ind_eq_increase + ...
          ind_eq_compare(k-l) - ind_eq_compare(k-l+1);
      end
      ind_eq_increase = ind_eq_increase / 5;
   end

   % display strategies once every l iterations:

   l = 1;
   if (mod(k,l) == 0)
      display_group_strategy(ind_eq_strategy,pop_groups,k)
   end

end

   plot_eq_compare(ind_eq_compare,k);

end

%%%%%%%%%%%%%%%%%%%%%%%%%%%%%%%%%%%%%%

function ind_strategy_out = update_strategy ...
(ind_strategy_in,pop_strategy_in,weights,...
alpha,beta,delta,kappa,theta,group)

%
%   Update individual distancing strategy
%
%   ind_strategy_in
%   -- current individual strategy, n x 1
%   pop_strategy_in
%   -- current population strategy, n x 1
%
%   alpha -- contact factors, n x 1
%   beta -- impact factors, n x 1
%
```

```
%   delta -- balancing parameters, n x 1
%
%   ind_strategy_out
%   -- updated individual strategy, n x 1
%
%
%   Zhijun Wu, 04/01/2024, Math Dept, Iowa State University
%

n = size(ind_strategy_in,1); M = size(pop_strategy_in,2);
k = group;

x = ind_strategy_in;
y = pop_strategy_in;

% pot_risk -- potential distancing risk;
% pop_risk -- distancing risk of the population;

omega = delta(1:n,k) .* alpha + ...
        (1 - delta(1:n,k)) .* beta;
pot_risk = 3.00 * omega ./ ...
        (1 + exp(-kappa .* (y(1:n,k) - theta)));

for l = 1 : M
    omega = delta(1:n,l) .* alpha + ...
    (1 - delta(1:n,l)) .* beta;
    pot_risk = pot_risk + omega ./ ...
    (1 + exp(-kappa .* (y(1:n,l) - theta)));
end

pop_risk = y(1:n,k)' * pot_risk;

   for i = 1 : n

       % strategy i has lower contact, i
       % ncrease its frequency

       if (pop_risk > pot_risk(i))
          if (x(i) < y(i,k))
             x(i) = x(i) + 0.9 * (y(i,k) - x(i));
          % * (pop_risk - pot_risk(i)) / pop_risk;
          else
             x(i) = x(i) + 0.1 * min(x(i)-y(i,k),1.0-x(i));
          % * (pop_risk - pot_risk(i)) / pop_risk;
          end
       end

       % strategy i has higher contact,
       % reduce its frequecy:

       if (pop_risk < pot_risk(i))
          if (x(i) > y(i,k))
             x(i) = x(i) - 0.9 * (x(i) - y(i,k));
          % * (pot_risk(i) - pop_risk) / pot_risk(i);
          else
             x(i) = x(i) - 0.1 * min(y(i,k)-x(i),x(i)-0.0);
          % * (pot_risk(i) - pop_risk) / pot_risk(i);
          end
       end

       % pot_risk at i is close to pop_risk,
       % adjust frequency to y(i):

       if (abs(pop_risk - pot_risk(i)) < 0.01)
          if (x(i) > y(i,k))
             x(i) = x(i) - 0.5 * (x(i) - y(i,k));
          end
          if (x(i) < y(i,k))
```

```
            x(i) = x(i) + 0.5 * (y(i,k) - x(i));
         end
      end

   end

ind_strategy_out = x / sum(x);

end

%%%%%%%%%%%%%%%%%%%%%%%%%%%%%%%%%%%%%%%%%%%%%%%%%%%

function plot_eq_compare (strategy_eq_compare,k)

plot(strategy_eq_compare(1:k),'-b','LineWidth',2);
hold on;

title(['Covergence to equilibrium <||x - x*||> \leq ',...
num2str(strategy_eq_compare(k),'%0.5f\n')],' ',...
'FontSize',16);
xlabel('Generations','FontSize',16,'FontWeight','bold');
ylabel('<||x - x*||>','FontSize',16,'FontWeight','bold');

ax = gca;
ax.XTick = unique(round(ax.XTick));

hold off;
pause;

end

%%%%%%%%%%%%%%%%%%%%%%%%%%%%%%%%%%%%%%%%%%%%%%%%%%

function display_group_strategy ...
(ind_strategy,pop_groups,k)

acts_0 = ...
[0 1 2 3 4 5 6 7 8 9 10 11 12 13 14 15 16 17 18 19 20];
sc = ["or","om","oc","ob","oy","og"]';

[n,m] = size(ind_strategy); M = size(pop_groups,2);

ind_average = sum (ind_strategy,2) / m;

plot(ind_average*112,'*k','MarkerSize',12,'LineWidth',1);
hold on;

for j = 1 : m
    for i = 1 : M
        if (pop_groups(j,i) == 1)
           l = i;
        end
    end
    plot(ind_strategy(1:n,j)*112,sc(l),...
    'MarkerSize',12,'LineWidth',1.5);
end

xticks(acts_0);

title(['Generation ',num2str(k),': ',...
'Strategies of Different Groups'],' ','FontSize',16);
xlabel('Social Activities','FontSize',16,...
'FontWeight','Bold');
ylabel('Active Time (Hours)','FontSize',16,...
'FontWeight','Bold');

plot(ind_average*112,'*k','MarkerSize',12,'LineWidth',1);
```

```
X = 1:1:20; Y = 0:1:20;
E20 = ones(20,1); E21 = ones(21,1);
X = E21 * X; Y = Y' * E20';

plot(X,Y,'-.c','LineWidth',1);

hold off;
pause;

end
```
