# Supplementary material for: Beyond six feet: The collective behavior of social distancing
Source: PLoS One. 2024 Sep 13;19(9):e0293489. doi: 10.1371/journal.pone.0293489 (PMC11398703; doi:10.1371/journal.pone.0293489)
Supplement: S1 Fig — (PDF) [file pone.0293489.s007.pdf]

```
function info = SI_plot_figures(i)
%
% This code prints the figures of the paper
% Beyond size feet: ...
%
% Fig 1, 2, 3, 4, 6 can be printed. Fig 5 is
% printed by simulation code 2.
%
% To print figure i, simply type:
%
% >SI_plot_figures(i);
%

if (i == 1)
   plot_fig_1; return;
end
if (i == 2)
   plot_fig_2; return;
end
if (i == 3)
   plot_fig_3; return;
end
if (i == 4)
   plot_fig_4; return;
end
if (i == 6)
   plot_fig_6; return;
end

disp('Do not know the number!');

end

%%%%%%%%%%%%%%%%%%%%%%%%%%%%%%%%%%%%%%%%%%%%%
% code for ploting figure 1:
%%%%%%%%%%%%%%%%%%%%%%%%%%%%%%%%%%%%%%%%%%%%%

function info = plot_fig_1 ()

f = figure(1);

k = 10; c = 0.5;
plot_logistic(k,c); hold;
x = c-0.03; y = s(x,k,c);
text(x-0.04,y,['\theta_i = ',num2str(c)],'FontSize',12,...
'FontWeight','Bold');

k = 10; c = 0.4;
plot_logistic(k,c);
x = c - 0.00; y = s(x,k,c);
text(x-0.04,y,['\theta_i = ',num2str(c)],'FontSize',12,...
'FontWeight','Bold');

k = 10; c = 0.6;
plot_logistic(k,c);
x = c - 0.06; y = s(x,k,c);
text(x-0.04,y,['\theta_i = ',num2str(c)],'FontSize',12,...
'FontWeight','Bold');

k = 10; c = 0.3;
plot_logistic(k,c);
x = c + 0.03; y = s(x,k,c);
text(x-0.04,y,['\theta_i = ',num2str(c)],'FontSize',12,...
'FontWeight','Bold');

k = 10; c = 0.7;
plot_logistic(k,c);
x = c - 0.09; y = s(x,k,c);
```

```
text(x-0.04,y,['\theta_i = ',num2str(c)],'FontSize',12,...
'FontWeight','Bold');


title(['Logistic Function: ',...
'\sigma_i(y_i) = 1/[1+e^{-\kappa_i(y_i-\theta_i)}]'],...
'FontSize',16,'FontWeight','Bold');
xlabel('y_i','FontSize',16,'FontWeight','Bold');
ylabel('\sigma_i','FontSize',16,'FontWeight','Bold');

text(0.1,0.85,'\kappa_i = 10','FontSize',16,...
'FontWeight','Bold');
hold;

end

function y = s(x,k,c)

y = 1 / (1 + exp(-k*(x-c)));

end

function info = plot_logistic (k,c)

x = 0:0.01:1;
y = 1 ./ (1 + exp(-k*(x - c)));
plot(x,y,'LineWidth',4);

end

%%%%%%%%%%%%%%%%%%%%%%%%%%%%%%%%%%%%%%%%%%%%
% code for plot_figure 2:
%%%%%%%%%%%%%%%%%%%%%%%%%%%%%%%%%%%%%%%%%%%%

function info = plot_fig_2 ()

X = ...
[14 14 14 14 7 7 7 7 4 4 4 4 2 2 2 2 1 1 1 1]';

Z = [ ...
9.1731 9.1731 7.7534 7.7534 ...
6.5075 5.3738 5.3738 6.5075 ...
5.0401 4.0397 4.0397 5.0401 ...
3.5060 3.5060 7.4943 8.2701 ...
3.7740 3.7740 2.9502 2.9502]';

Y = ...
[5 5 3 3 5 3 3 5 5 3 3 5 4 4 16 20 6 6 4 4]';

f = figure(1);

plot_activity(X,Y,Z);

end

function info = plot_activity (x,y,z)

%for i = 1 : 20
%X(2*i-1)=x(i);X(2*i)=x(i); AX(2*i-1)=i-1+0.5;AX(2*i)=i+0.5;
%end
%for i = 1 : 20
%Y(2*i-1)=y(i);Y(2*i)=y(i); AY(2*i-1)=i-1+0.5;AY(2*i)=i+0.5;
%end
%for i = 1 : 20
%Z(2*i-1)=z(i);Z(2*i)=z(i); AZ(2*i-1)=i-1+0.5;AZ(2*i)=i+0.5;
%end

%plot(AX,X,AY,Y,AZ,Z,'LineWidth',6);
```

```
%hold;

a = 1:1:20; a = a';
plot(a,x,'o','MarkerSize',12,'LineWidth',3,'Color','Blue');
hold;
plot(a,y,'o','MarkerSize',12,'LineWidth',3,'Color','Red');
plot(a,z,'+','MarkerSize',12,'LineWidth',3,'Color','#EDB120');

acts_0 = [0 1 2 3 4 5 6 7 8 9 10 11 12 13 14 15 16 17 18 19 20];
acts_1 = {'  ';'reading/watching TV';'work at home';'hiking';...
        'gardening';'stay with family';'grocery shopping';...
        'hospitals';'visit friends';'restaurant dinning';...
        'shopping malls';'take buses';'go to churches';...
        'sports';'concerts';'schools';'workplaces';...
        'large gathering';'attend bars';'air traveling';...
        'movie theaters'};

xticks(acts_0); xticklabels(acts_1); xtickangle(45);

axis([0 21 0 20]);

title(["Equilibrium Strategies w/o Social Distancing"," "],...
'FontSize',16);
xlabel('Social Activities','FontSize',16,'FontWeight','Bold');
ylabel('Active Time (Hours)','FontSize',16,'FontWeight','Bold');



text(7,17,['o ','\delta_i = 1'],'FontSize', 16, 'Color',...
'Blue','FontWeight','Bold');
text(7,14,['o ','\delta_i = 0'],'FontSize', 16, 'Color',...
'Red','FontWeight','Bold');
text(7,11,['+ ','\delta_i = 0.5'],'FontSize', 16, 'Color',...
'#EDB120','FontWeight','Bold');

hold;

end


%%%%%%%%%%%%%%%%%%%%%%%%%%%%%%%%%%%%%%%%%%%%
% code for plot_figure 3:
%%%%%%%%%%%%%%%%%%%%%%%%%%%%%%%%%%%%%%%%%%%%

function info = plot_fig_3 ()

m = 200; K = 6; b = 0.30;

plot_network (m, K, b);

end

function info = plot_network (m,K,b)

rng ('default');

soc_net = small_world(m,K,b);
G=graph(soc_net,'omitselfloops');

step=2*pi/m; phi=step:step:2*pi;
x=10*cos(phi); y=10*sin(phi);

clf;
f = figure(1);
f.Position(3) = 1120;

subplot(1,2,1);
plot(G,'XData',x,'YData',y,'LineWidth',1,'MarkerSize',4);
```

```
title(['Small World Network: ','m = ',num2str(m),...
', K = ',num2str(K),', b = ',num2str(b)]," ",'FontSize',...
16,'FontWeight','Bold')

subplot(1,2,2);
histogram(sum(soc_net));

xlabel('Degree of Nodes','FontSize',16,'FontWeight','Bold');
ylabel('Number of Nodes','FontSize',16,'FontWeight','Bold');
title("Distribution of Degrees of Nodes in Network"," ",...
'FontSize',16,'FontWeight','Bold')

end

function soc_net = small_world (N,K,b)

%
% Generation of Small-World Population Network
%
% N -- population size
% K -- degree of connection, even number
% b -- randomness parameter, [0,1]
%
% soc_net -- adjacency matrix of population network
%
% Zhijun Wu, 01/15/2022, Math Dept, Iowa State University
%

soc_net = zeros(N);

for i = 0 : N-1
    k = 1;
    while (k <= K/2)
        j = mod(i+k,N);
        soc_net(i+1,j+1) = 1;
        soc_net(j+1,i+1) = 1;
        k = k +1;
    end
    k = 1;
    while (k <= K/2)
        j = mod(i-k,N);
        soc_net(i+1,j+1) = 1;
        soc_net(j+1,i+1) = 1;
        k = k + 1;
    end
end

for i = 0 : N-1
    k = 1;
    while (k <= K/2)
        j = mod(i+k,N);
        if (soc_net(i+1,j+1) == 1)
           l = floor(N*rand);
           while (l == i || soc_net(i+1,l+1) == 1)
              l = floor(N*rand);
           end
           if (rand <= b)
              soc_net(i+1,l+1) = 1;
              soc_net(l+1,i+1) = 1;
              soc_net(i+1,j+1) = 0;
              soc_net(j+1,i+1) = 0;
           end
        end
        k = k +1;
    end
end
```

```
%writematrix(soc_net,'soc_net.dat','Delimiter',' ');

end


%%%%%%%%%%%%%%%%%%%%%%%%%%%%%%%%%%%%%%%%%%%%
% code for plot_figure 4:
%%%%%%%%%%%%%%%%%%%%%%%%%%%%%%%%%%%%%%%%%%%%

function plot_fig_4 ()

x = [4.5984e-03 5.2092e-04      1.0790e-04      ...
1.2446e-04      7.1508e-05      5.9851e-05];
y = [3.7795e-03 8.5057e-05      2.1823e-04      ...
6.9527e-05      5.0005e-05      5.6804e-05];
z = [4.7625e-03 6.3291e-04      1.6536e-04      ...
7.7867e-05      6.7857e-05      6.5584e-05];

clf;
f = figure(1);
f.Position(3) = 1120;

subplot(1,2,1);
plot_convergence_1(x,y,z);

x = [4.5859e-03 9.6347e-04      6.5605e-05      ...
7.3907e-05      5.0429e-05      9.3284e-05];
y = [4.0299e-03 2.8287e-04      6.0179e-05      ...
6.9300e-05      4.7988e-05      4.2705e-05];
z = [3.7795e-03 8.5057e-05      2.1823e-04      ...
6.9527e-05      5.0005e-05      5.6804e-05];

subplot(1,2,2);
plot_convergence_2(x,y,z);

end

function info = plot_convergence_1 (x,y,z)

k = 1 : 1 : 6;

plot(k,x,'o-','Color','Red','LineWidth',4,'MarkerSize',12);
hold;
plot(k,y,'o-','Color','#EDB120','LineWidth',4,'MarkerSize',12);
plot(k,z,'o-','Color','Blue','LineWidth',4,'MarkerSize',12);

title('Convergence of Network Simulation','FontSize',16);
xlabel('Neighborhood Sizes','FontSize',16,'FontWeight','Bold');
ylabel('Convergence Errors','FontSize',16,'FontWeight','Bold');

xticks([2 3 4 5 6]); axis([2 6 0.0000 0.0007]);

text(3.0,5.5e-4,'b = 0.30','FontSize', 16, 'Color','Blue',...
'FontWeight','Bold');
text(4.5,5.5e-4,['-o-  ','\delta_i = 0.0'],'FontSize', 16,...
'Color','Red','FontWeight','Bold');
text(4.5,4.5e-4,['-o-  ','\delta_i = 0.5'],'FontSize', 16,...
'Color','#EDB120','FontWeight','Bold');
text(4.5,3.5e-4,['-o-  ','\delta_i = 1.0'],'FontSize', 16,...
'Color','Blue','FontWeight','Bold');

hold;

end

function info = plot_convergence_2 (x,y,z)

k = 1 : 1 : 6;
```

```
plot(k,x,'o-','Color','Red','LineWidth',4,'MarkerSize',12);
hold;

plot(k,y,'o-','Color','#EDB120','LineWidth',4,'MarkerSize',12);
plot(k,z,'o-','Color','Blue','LineWidth',4,'MarkerSize',12);

title('Convergence of Network Simulation','FontSize',16);
xlabel('Neighborhood Sizes','FontSize',16,'FontWeight','Bold');
ylabel('Convergence Errors','FontSize',16,'FontWeight','Bold');

xticks([2 3 4 5 6]); axis([2 6 0.0000 0.0010]);

text(3.0,8.0e-4,'\delta_i = 0.50','FontSize', 16, ...
'Color','Red','FontWeight','Bold');
text(4.5,8.0e-4,['-o-  ','b = 0.10'],'FontSize', 16, ...
'Color','Red','FontWeight','Bold');
text(4.5,6.55e-4,['-o-  ','b = 0.20'],'FontSize', 16, ...
'Color','#EDB120','FontWeight','Bold');
text(4.5,5.1e-4,['-o-  ','b = 0.30'],'FontSize', 16, ...
'Color','Blue','FontWeight','Bold')
hold;

end

%%%%%%%%%%%%%%%%%%%%%%%%%%%%%%%%%%%%%%%%%%%%
% code for plot_figure 6:
%%%%%%%%%%%%%%%%%%%%%%%%%%%%%%%%%%%%%%%%%%%%

function plot_fig_6 ()

g1 = [1.6697e-02   1.0632e-02   ...
7.2883e-03   5.0961e-03   4.0525e-03];
g2 = [1.3564e-02   1.0463e-02   ...
8.2715e-03   5.6613e-03   4.6434e-03];
g3 = [2.0940e-02   1.5410e-02   ...
1.2393e-02   1.0566e-02   8.7432e-03];
g4 = [1.4089e-02   1.0992e-02   ...
8.9342e-03   7.2468e-03   5.8082e-03];

clf;
f = figure(1);
f.Position(3) = 1120;

subplot(1,2,1);
plot_lead_1(g1,g2,g3,g4);

g1 = [1.1352e-03   1.4115e-03   ...
1.4053e-03   1.3578e-03   1.4287e-03];
g2 = [1.4289e-03   1.5554e-03   ...
1.6113e-03   1.6073e-03   1.5877e-03];
g3 = [2.9661e-03   3.1150e-03   ...
3.3167e-03   3.4776e-03   3.6229e-03];
g4 = [2.4610e-03   2.6128e-03   ...
2.6331e-03   2.6792e-03   2.6836e-03];

subplot(1,2,2);
plot_lead_2(g1,g2,g3,g4);

end

function info = plot_lead_1 (g1,g2,g3,g4)

k = 1 : 1 : 5;

plot(k,g1,'o-','Color','Red','LineWidth',4,'MarkerSize',12);
hold;
plot(k,g2,'o-','Color','Magenta','LineWidth',4,'MarkerSize',12);
```

```
plot(k,g3,'o-','Color','Cyan','LineWidth',4,'MarkerSize',12);
plot(k,g4,'o-','Color','Blue','LineWidth',4,'MarkerSize',12);

title('Convergence of Leader/Follower Simulation','FontSize',16);
xlabel('%Leaders','FontSize',16,'FontWeight','Bold');
ylabel('Convergence Errors','FontSize',16,'FontWeight','Bold');

axis([0.5 5.5 3.0e-3 2.5e-2]);
xticks([0.5 1 2 3 4 5 5.5]);
xticklabels({' ';'10%';'20%';'30%';'40%';'50%';' '});

text(2.5,0.022,'<1>','FontSize', 16, ...
'Color','Red','FontWeight','Bold');
text(4,0.022,['-o-  ','g1'],'FontSize', 16, ...
'Color','Red','FontWeight','Bold');
text(4,0.020,['-o-  ','g2'],'FontSize', 16, ...
'Color','Magenta','FontWeight','Bold');
text(4,0.018,['-o-  ','g3'],'FontSize', 16, ...
'Color','Cyan','FontWeight','Bold');
text(4,0.016,['-o-  ','g4'],'FontSize', 16, ...
'Color','Blue','FontWeight','Bold');

hold;

end

function info = plot_lead_2 (g1,g2,g3,g4)

k = 1 : 1 : 5;

plot(k,g1,'o-','Color','Red','LineWidth',4,'MarkerSize',12);
hold;
plot(k,g2,'o-','Color','Magenta','LineWidth',4,'MarkerSize',12);
plot(k,g3,'o-','Color','Cyan','LineWidth',4,'MarkerSize',12);
plot(k,g4,'o-','Color','Blue','LineWidth',4,'MarkerSize',12);

title('Convergence of Leader/Follower Simulation','FontSize',16);
xlabel('%Leaders','FontSize',16,'FontWeight','Bold');
ylabel('Convergence Errors','FontSize',16,'FontWeight','Bold');

axis([0.5 5.5 1.0e-3 0.76e-2]);
xticks([0.5 1 2 3 4 5 5.5]);
xticklabels({' ';'10%';'20%';'30%';'40%';'50%';' '});

text(2.0,6.7e-3,'<2>','FontSize', 16, ...
'Color','Red','FontWeight','Bold');
text(3.5,6.7e-3,['-o-  ','g1'],'FontSize', 16, ...
'Color','Red','FontWeight','Bold');
text(3.5,6.1e-3,['-o-  ','g2'],'FontSize', 16, ...
'Color','Magenta','FontWeight','Bold');
text(3.5,5.5e-3,['-o-  ','g3'],'FontSize', 16, ...
'Color','Cyan','FontWeight','Bold');
text(3.5,4.9e-3,['-o-  ','g4'],'FontSize', 16, ...
'Color','Blue','FontWeight','Bold');

hold;

end
```
