# Supplementary material for: Beyond six feet: The collective behavior of social distancing
Source: PLoS One. 2024 Sep 13;19(9):e0293489. doi: 10.1371/journal.pone.0293489 (PMC11398703; doi:10.1371/journal.pone.0293489)
Supplement: Attachment — Submitted filename: Response to Reviewers.pdf [file pone.0293489.s008.pdf]

Response letter for manuscript PONE-D-23-33255

Beyond six feet: The collective behavior of social distancing

submitted to PLOS ONE for publication

Dear Editor,

I would like to thank the reviewers for carefully reading my paper and providing valuable comments and suggestions. They are among the best reviews I have ever received on my papers. They are critical but sincere and motivated me to read more related work, especially in behavioral sciences, and helped me to revise and improve the paper substantially.

The reviewers' concerns on the work, its theoretical assumptions and experimental bases, are all legitimate. It is partially due to the complex nature of social distancing, and partially due to the limit of my knowledge in social sciences as well as health sciences research. I am new to this field and will hopefully be able to learn and improve.

I am now trying to make some substantial changes in the work as well as the presentation of the paper. I have reformulated the risk assessment function $p_i(y)$ using a nonlinear logistic function. This function increases slowly at the beginning, picks up the speed after a certain threshold, and slows down again in the end, which presumably corresponds better than a linear function to the increase of the distancing risk in a given activity as the population in the activity increases. With this change, I have revised all my codes, and reproduced and documented all the computation results. The tables and graphs in **Supplementary Information** are all updated accordingly. So are the tables and graphs in the main text and the proofs in **Appendix**.

The complicated dependence of social distancing on various social, economic as well as health influences may still be oversimplified even with this change, but hopefully it will provide more flexibilities to model the social distancing behavior more accurately.

New contents are added to the paper. Many places are revised with hopefully better descriptions and interpretations. In particular, I have discussed the theories and experiments in behavioral sciences including the theory of planned behavior and the health brief models, their shared principles with the game model, and the insights that can be learned from behavioral sciences into possible improvements on the game model. I have rewritten several pages in **Results** and **Methods** on the estimation of the parameters in the risk assessment functions. The estimates are not based on real data, but are extracted from reasonable assumptions on how people would do for complete or free of social distancing, which hints a possible path to determining the parameters in practice. I have also taken the reviewers' suggestions and removed several numerical tables and replaced them with more accessible graphs. There are now total six figures in the paper, all regenerated except for Fig 3.

The following are my more detailed responses to the reviewers' comments and the corresponding changes I have made in the paper. Attached please find the revised paper with new contents and changes highlighted in blue color. A copy of the paper without highlights is also submitted.

Thank you all very much!

Sincerely,

Zhijun Wu

Reviewer #1's General Comments:

The manuscript "Beyond six feet: The collective behavior of social distancing" presents a game-theoretic model for analyzing and simulating distancing strategies during infectious disease outbreaks. The model introduced is sound; the research has been conducted comprehensively, thoroughly, and systematically; the results are supported by detailed mathematical proofs and derivations as well as detailed descriptions of the simulations; and the manuscript is well-structured and well-written. The findings give interesting and valuable insights into the modeling of distancing strategies in game-theoretic scenarios of disease spread. The approach of starting with a highly abstract, simplified version of the model and then gradually introducing more complexity and details stands out to me, shows the rigor of the author, and enables me to understand the increasingly complex dynamics.

Response to Reviewer #1's General Comments:

Thanks for carefully reading the paper and providing valuable comments and suggestions! They have motivated me to read more related papers, especially those in behavioral sciences, and helped me to revise and improve my paper substantially.

The concerns on the work, its theoretical assumptions and experimental bases, are all legitimate. It is partially due to the complex nature of social distancing, and partially due to the limit of my knowledge in social sciences as well as health sciences research. I am new to this field and will hopefully be able to learn and improve.

I am now trying to make some substantial changes in the work as well as the presentation of the paper. I have reformulated the risk assessment function $p_i(y)$ using a nonlinear logistic function. This function increases slowly at the beginning, picks up the speed after a certain threshold, and slows down again in the end, which presumably corresponds better than a linear function to the increase of the distancing risk in a given activity as the population in the activity increases. With this change, I have revised all my codes, and reproduced and documented all the computation results. The tables and graphs in **Supplementary Information** are all updated accordingly. So are the tables and graphs in the main text and the proofs in **Appendix**.

The complicated dependence of social distancing on various social, economic as well as health influences may still be oversimplified even with this change, but hopefully it will provide more flexibilities to model the social distancing behavior more accurately.

New contents are added to the paper. Many places are revised with hopefully better descriptions and interpretations. In particular, I have discussed the theories and experiments in behavioral sciences including the theory of planned behavior and the health brief models, their shared principles with the game model, and the insights that can be learned from behavioral sciences into possible improvements on the game model. I have rewritten several pages in **Results** and **Methods** on the estimation of the parameters in the risk assessment functions. The estimates are not based on real data, but are extracted from reasonable assumptions on how people would do for complete or free of social distancing, which hints a possible path to determining the parameters in practice. I have also taken the reviewers' suggestions and removed several numerical tables and replaced them with more accessible graphs. There are now total six figures in the paper, all regenerated except for Fig 3.

The following are my more detailed responses to Review #1's comments and concerns and the corresponding changes I have made in the paper.

Reviewer #1's Comment #1:

In the current version of the manuscript, the model lacks a theoretical foundation in the behavioral

sciences. However, without proper grounding in behavioral theory, this research remains a modeling exercise. Starting points for health behavioral theories that consider risk perception could be the "Theory of Planned Behavior" (Godin & Kok, 1996) or the Health Belief Model (Green & Murphy, 2014). Empirical support could be found in reviews by Bish & Michie (2010) and Leppin & Aro (2009). Another starting point, to highlight the importance of considering risk perception in infectious disease modeling, could be reference 45 in the presented manuscript.

Response to Reviewer #1's Comment #1:

The theoretical foundation is indeed a great concern of this work. The original idea behind the work was motivated by the success of the game model on vaccine acceptance by Bauch and Earn 2004. In general, game theory has been applied to analyzing animal behaviors such as the evolution of social cooperation since the seminar paper on "The logic of animal conflict" by Smith and Price was published in 1973. It would certainly be interesting to see if social distancing can be modeled as a game, and its conflict yet cooperative nature can be revealed as such. However, human behavior is indeed much more complex than animal behavior in general. It is a social act, an economic act, as well as an act of certain intent. The subject indeed has to be studied in a complex social context. I appreciate very much that the reviewer points this out and provides me with valuable references to research on human behaviors and in particular, health behaviors in behavioral sciences.

I have read a few papers on human behavior as suggested by the reviewer, and listed their work in literature reviews in **Introduction**. I have also discussed the theory of planned behavior and the health belief models and how my work can be related to these theories in **Discussion**. More specifically, in **Introduction**, the following paragraph is revised with the modified sentences highlighted in blue color:

"Research on social distancing has surged since the outbreak of the COVID-19 pandemic, providing a wealth of knowledge and experience on non-pharmaceutical measures for preventing epidemics from spreading. However, most of these studies are about the influences of public policies, economic concerns, or cultural differences on social distancing, but not particularly about social distancing as a human behavior or the collective behavior of a population for that matter, as pointed out in a recent article by Vardavas et al. 2021 [60]. In fact, human responses to and influences on public health measures such as mask wearing, testing, and vaccination have long been investigated in behavioral sciences based on the theory of planned behavior [61,62] and the health belief models [63,64], with great insights into how health behaviors may be perceived and carried out at both individual and population levels."

The following two paragraphs are added to **Discussion**:

"Social behaviors including health-related behaviors have been studied for long time in behavioral sciences, most notably, in the theory of planned behavior [61,62] and the health belief models [63,64]. The work in this paper is not directly based on these theories but is consistent with their general principles. In the health belief models, the individual responses to health concerns are considered to be based on the outcomes of the assessments on the perceived susceptibility to the ill, perceived severity of the ill, perceived barriers to take actions, and perceived benefits of behavioral changes [63,64]. The estimates on the distancing risks in the game model can be considered as the results from similar assessments, although not explicitly correlated to them. Since the game model is focused more on the strategies of participating in social activities, the risk assessments depend more on the selected activities as well as the distribution of the population over the activities."

"In the theory of planned behavior, several critical components are identified for social behaviors, namely, the attitude, the social norms, and the perceived behavior control. They affect the attentions or motivations which then in turn determine the actions. In this work,

these factors have not been fully considered. The individuals are assumed implicitly to be in total control of switching among different distancing actions, which may not be true in general according to the studies on individual responses to public health measures [62,63]. It would be interesting to take into account the influences on distancing decisions from the behavioral determinants suggested by the theory of planned behavior. For example, individuals with different attitudes toward social distancing may be considered as different population groups of different risk perceptions; certain control factors such as the ability to participate in certain activities such as remote work may need to be considered before a distancing action can be carried out even if it is justified with the risk assessment."

Reviewer #1's Comment #2:

In addition to the previous comment, the author claims that "Work on modeling social distancing [... had] little specifics on how the distancing activities are carried out and how certain distancing patterns or levels are achieved." I would agree that this is an emerging field. However, there is recent literature that can be referenced here. For example, Nunner et al. (2022) showed in a model study with risk perception that clusters of individuals that reduce social contacts homogeneously can mitigate disease spread. The model is based on a game-theoretic model of network formation by Jackson (2008) with comparable considerations (as described in lines 42-49). Another example would be Woike et al. (2022), who explore the effectiveness of behavioral interventions in controlling the spread of infectious diseases through simulations and experiments. A comparison of the results with the findings in these studies would provide an interesting extension of the discussion.

Response to Reviewer #1's Comment #2:

Thanks for pointing this out. I have found the work by Woike et al. 2022 to be particularly interesting. In their work, the health behavior is also considered as a game. Each individual has to make a behavioral decision among conflicting interests, which shares the same nature of the distancing game. In addition, the experimental game carried out in their work provides helpful insights into how people engage in health activities and react to public health policies under various possible conditions. With that I have added a whole paragraph to discuss their work in **Discussion**:

"Another interesting piece of game theoretic work on the health behaviors is done by Woike et al. 2022 [117]. An experimental game is carried out in [117] in two small but real populations, where the individuals are given choices to take low-risk, low-reward vs high-risk, high-reward protections from an assumed epidemic. It turns out that in several rounds of the game, a very high percentage of individuals tend to take the high-risk actions although highly likely to be infected and no rewards. The game is then conducted in several possible scenarios, where the players are provided with information on the development of the epidemic, the actions of other participants, healthcare promotions, outcomes of infections, etc. The results from the simulated game show how the individual actions are influenced by the provided information. While social distancing activities are complex and hard to survey, the simulated game in [117] can be quite helpful for not only providing insights into the individual behavioral responses to epidemics but also improving the existing epidemiological models including the model proposed in this work."

Jackson 2008 is cited in the discussion on social networks in **Introduction**. The paper by Nunner 2022 along with Singh 2020 and Kadelka 2023 is cited in **Discussion** for future exploration on possible influences of network structure on social distancing.

Reviewer #1's Comment #3:

The discussion is mostly a (well-written) summary of the paper. However, it lacks a more explicit discussion of the results in the context of the literature and the practical implications of the research for epidemic intervention and public health policy. Some previous comments might be useful for a more refined discussion.

Response to Reviewer #1's Comment #3:

As in the response to Comment #2, I have now added two paragraphs in **Discussion** to discuss the theory of planned behavior and the health belief models and how my work relates to them. I have also added one paragraph on the work by Woike et al. 2022 which, similar to my work, also tries to reveal a game nature of health behaviors, although experimentally. In addition, I have added the following paragraph in the first page of **Discussion** to further stress how the parameters, the contact factors and impact factors, in the assessment functions for the distancing risks are determined. Although not based on real data, they are extracted from reasonable assumptions on how people would do for complete or free of social distancing, which hints a possible path to determining the parameters in practice:

"The model is built by referring to two possible population states where the distancing risks of a set of social activities are assessed. First, a complete social distancing state is assumed, and the corresponding risk factors are extracted as the contact factors. Then, a free of social distancing state is assumed, and the corresponding risk factors are derived as the impact factors. The contact factors are correlated with but not equivalent to the contact rates. They are so named as in a complete social distancing state, the close social contacts are perceived as the main social risks to reduce. Similarly, the impact factors are not directly related to the social or economic costs of the activities. They are so named as in a free of social distancing state, the negative social or economic impacts are the main concerns to minimize. By combining the two sets of parameters, a general set of risk factors can then be defined for a population in between the two extremum states."

The follow paragraph in **Discussion** is also modified to further explain the results for heterogeneous populations and their implications.

"The extension of the distancing game to populations with multiple population groups in **Results – In heterogeneous populations** allows each population group to have its own assessment on distancing risks and hence its own distancing strategy. In such a game, the individuals take into account the contributions to their distancing risks from all population groups while giving more weights to those from their own groups, as they are supposed to interact more with the individuals in their own groups than those in other groups. In theory, the game converges to an equilibrium strategy, with each group having a strategy as if it plays the game alone as justified in **Appendix – Games with multiple social groups**. This property is further observed in the simulation when the population is spread on a social network. It reveals a critical condition under which multiple social groups compete yet maintain their own independent strategies. It applies well to social distancing activities in heterogeneous populations. Future work along this line may be extended to the effects on social distancing from the homophilic structure of the population and the clustering structure of the social network as studied in recent work in [113-115]."

Reviewer #1's Comment #4:

Presentation of the results regarding tables and figures should be improved.
(a) I find Table 1 difficult to read. A vertical layout with activities written out would improve accessibility.
(b) Table 2 shows the same information as Figure 1, while Figure 1 is more accessible. Further-

more, Figure 1 should not use lines because they suggest continuous data on the x-axis. A plot in the style of Figure 3 would be more fitting.
(c) Tables 3 and 4 might be difficult to access for a broader interdisciplinary audience, such as the readership of PLOS ONE. Providing some more intuition about the Euclidian norm would help.
Suggestion: A plot that shows the combination of results for all scenarios, in the style of Figure 3, would enable a better and more intuitive comparison of the results across the different scenarios and relax the issues above.

Response to Reviewer #1's Comment #4:

For (a), a double line is used to separate the values for activities 1-10 and activities 11-20 so that the table is easier to read. The following paragraphs are also added to further explain the contents in the table:

"Table 1 shows the values of α_i and β_i estimated for a small set of so-called commonly attended social activities or CASA activities for short. These activities are assumed to be typical in daily small town lives in North America and are grouped into 20 general categories for the testing and simulation purposes in this work. In practice, they can certainly be extended to a much larger set of more specific and refined activities."

"The first eight CASA activities include 1. reading/watching TV, 2. work at home, 3. hiking, 4. gardening, 5. stay with family, 6. grocery shopping, 7. go to hospitals, 8. visit friends. They have low contact rates and hence relatively low contact factors. The last eight CASA activities include 13. watch sports, 14. attend concerts, 15. go to schools, 16. go to workplaces, 17. large gathering, 18. go to bars or night clubs, 19. air traveling, 20. go to movie theaters. They have high contact rates and hence relatively high contact factors. In between, there are also four frequently participated CASA activities, 9. restaurant/cafeteria dinning, 10. go to shopping malls, 11. take buses, 12. go to churches. Their values of contact factors are at a moderate level."

"The values for the impact factors are not necessarily negatively correlated with the values of the contact factors. They vary in a small range except for activities 15. go to schools and 16. go to workplaces whose impact factors are quite small. The smaller the impact factor, the less negative social or economic impact in the activity or in other words, the more socially or economically favorable. For example, although in the same level of contact factors, activities 3. hiking and 4. gardening have relatively larger impact factors than activities 1. reading/watching TV and 2. work at home which seem to be more socially or economically favorable. On the other hand, although activities 17. large gathering and 18. go to bars or night clubs have relatively large contact factors, their impact factors are smaller than most of the activities because of their high social demands. The impact factors for activities 15. go to schools and 16. go to workplaces are among the smallest, agreeing with the fact that they are certainly two of the most important social and economic activities in modern human lives."

For (b), since a nonlinear function is now introduced to define the risk assessment function $p_i(y)$, a new figure is plotted as Fig 1 to demonstrate the behavior of the function, and the original Fig 1 becomes Fig 2, where the results are also changed. The lines in the original figure are changed to circles. Table 2 is removed, since it is not so accessible and shows the same results as in Fig 2. More detailed explanations on Fig 2 are added as follows:

"Now, let $w_i = \delta_i\alpha_i + (1-\delta_i)\beta_i$, $0 \leq \delta_i \leq 1$. A general set of risk factors can be obtained with δ_i called a severity parameter. If $\delta_i = 1$ for all i, $w_i = \alpha_i$. If $\delta_i = 0$ for all i, $w_i = \beta_i$. If $0 < \delta_i < 1$, w_i determines an equilibrium strategy x^* and hence y^* that corresponds to a population state between complete and free of social distancing. Based on general theory for population games, given $p_i(y) = w_i\sigma_i(y_i)$, if $w_i > 0$ for all i, an equilibrium strategy x^* for the

distancing game can be obtained with $x_i^* = \sigma_i^{-1}(\lambda/w_i),\ i = 1, \ldots, n$, assuming $x_i^* > 0$ for all i, where λ is a constant such that $\Sigma_i\, \sigma_i^{-1}(\lambda/w_i) = 1$ (details in **Equilibrium strategies and stabilities** in **Methods**)."

"For convenience, let the same logistic function σ_i with $\kappa_i = 10$ and $\theta_i = 0.5$ be used for all the activities in Table 1. Then, the equilibrium strategies of the distancing game for this set of activities can immediately be computed for some fixed value of δ_i. Fig 2 illustrates the contrast among three of these equilibrium strategies with $\delta_i = 1$, 0, or 0.5 for all i, corresponding to the games for complete social distancing, no social distancing, or partially social distancing, respectively. For complete social distancing (blue circles, $\delta_i = 1$) when close social contacts are supposed to be reduced the most, for the first eight CASA activities whose contact rates are low, the participating frequencies are high, while for the last eight activities whose contact rates are high, the participating frequencies are low. On the other hand, if free of social distancing (red circles, $\delta_i = 0$) when negative social or economic impacts of social distancing are contained the most, for the first eight activities, the participating frequencies are much lower than those for complete social distancing, while for the last eight activities, the participating frequencies are much higher. In between when both contacts and impacts are concerned (brown plus signs, $\delta_i = 0.5$), for the first eight activities, the participating frequencies are still higher than those free of social distancing, but not as high as those for complete social distancing, and for the last eight activities, the participating frequencies are certainly lower than those free of social distancing, but not as low as those for complete social distancing."

For (c), original Table 3 and Table 4 are replaced by Fig 4 and Fig 6, respectively, with multiple lines combined together to demonstrate the convergence behaviors of the simulation in different scenarios, as suggested by the reviewer. The following two paragraphs are modified to explain Fig 4 in **Results – In small world social networks**:

"The simulation runs in multiple iterations, each named as a generation. In each generation, every individual gets a chance to update his/her strategy followed by an adjustment on the population strategy. The simulation ends when every individual strategy x becomes very close to the equilibrium strategy x^* of the game. The closeness is measured by a so-called Euclidean distance $\|x - x^*\|$ between the two strategies, $\|x - x^*\| = \sqrt{\Sigma_i (x_i - x_i^*)^2}$. The simulation is considered to be converged if the average Euclidean distance $<\|x - x^*\|>$ between x and x^* in the whole population is smaller than a prescribed small number, say 10^{-4}."

......

"Fig 4 demonstrates the network simulation results for two typical scenarios: In Fig 4 (a), the convergence results are shown for the game with $\delta_i = 0.0, 0.5, 1.0$ and $k = 2, 3, 4, 5, 6$ while b is fixed to 0.30. In Fig 4 (b), the results are shown for the game with $b = 0.10, 0.20, 0.30$ and $k = 2, 3, 4, 5, 6$ while δ_i is fixed to 0.5. In both cases, for different values of δ_i and b, the game converges for all different neighborhood sizes greater than or equal to 2. The accuracy for $k = 2$ is not very high but acceptable. It improves as k becomes larger. When b is fixed to 0.30 but k is changed from 1 to 2, 3, 4, 5, 6, the average number of neighbors for each individual changes from 7 to 29, 116, 425, 1187, 1897. The last number agrees with the general consensus on small-world social networks where almost every pairs of individuals can be found connected with up to more or less 6 consecutive links [105-107]. ... "

The following paragraphs are modified to explain Fig 6 in **Results – By following the leaders**.

"The first set of results <1> in Fig 6 (a) is obtained with a follow-the-crowd strategy if a follower cannot find a group leader among his/her closest neighbors. When there is a high percentage of leaders in the population ($\geq 30\%$), the game converges to the equilibrium strategies in reasonable accuracies for all population groups. If there are lower than 30% leaders in the

population, the convergence becomes less accurate, when most of the followers are not able to find a group leader in their neighborhood, and there is a disadvantage by simply following the crowd."

"The second set of results <2> in Fig 6 (b) is obtained with a make-own-decision strategy if a follower cannot find a group leader among his/her closest neighbors. Similar to the previous case, when there is a high percentage of leaders in the population ($\geq 30\%$), the game converges to the equilibrium strategies in reasonable accuracies for all population groups. In contrast to the previous case, when the percentage of leaders are less than 30%, the convergence remains to be as accurate, because when there are fewer leaders, more followers start making their own decisions, which can be even better than following the leaders."

Reviewer #1's Comment #5:

Why was $k = 3$ chosen for the networks in the heterogeneous and leader-follower scenarios? Earlier (line 207), a setting of $k = 6$ was supported by literature. Although the Milgram experiment has received much criticism over the years, it provides reasoning. Context-related research, such as the POLYMOD study by Mossong et al. (2008) or similar work on social encounter networks in Great Britain by Danon (2013), is more suitable to defend degree settings. The former would also provide more solid empirical footing for the decision to distinguish contact numbers by age.

Response to Reviewer #1's Comment #5:

Thanks for suggesting the two references. Several new references are added in **Introduction** when the issues with the general game model are brought up. Mossong 2008 and Danon 2012 are cited for contact patterns and social encounter networks. Wolfe 2021 and Zimmermann 2022 are cited for age differences in the perception on health protection. Davis 2023 and Pangallo 2024 are cited for economic differences. Olsen 2020, Hills 2021, and Akfirat 2023 are cited for the willingness of engaging social distancing and the leadership role in health prevention.

As for $k = 3$, k is the neighborhood size for simulation. A neighborhood of size k for a given node is the set of nodes that connect with the given node in maximum k consecutive links. If $k = 6$, all the nodes in the network may be included in every neighborhood according to the small world theory. That is the case almost equivalent to the general distancing game when each individual node plays the game against all other nodes. In order to see a nontrivial case, a smaller neighborhood size than 5 or 6 would be better. Therefore, $k = 3$ is used to demonstrate how the simulation performs on a network when every individual only interacts with a restricted number of neighbors. In any case, the simulation is carried out for all $k = 1, 2, 3, 4, 5, 6$. The results are all documented and reported in **Simulation results 1, 2, 3** in **Supplementary Information**. The cases for $k = 3$ are selected as sample results for demonstration purposes only in the main text.

Reviewer #1's Comment #6:

The "following the leaders" parts (Results and Methods) require refinement and further elaboration:
(a) "A small number of leaders" is stated multiple times (e.g., line 90, line 289). However, this number can actually go up to 60% (line 561).
(b) How were the leaders selected?
(c) How far can followers be from leaders, while still considering them to be leaders? (line 555-557)
(d) What are the "multiple population groups" (line 555), how are they defined, and how large are they?
(e) How is it decided whether an individual makes his/her own decision as a regular player or

follows the crowd (lines 558-559)?

Response to Reviewer #1's Comment #6:

(a) It is indeed not "small". The phrase is now changed to "a certain number of leaders."
(b) The leaders are randomly assigned at the very beginning of the simulation.
(c) An individual looks for a leader only in his/her group among his/her closest neighbors.

The following paragraph in **Results – By following the leaders** is modified:

"In order to incorporate the leadership factor into the distancing model, a certain number of individuals are designated randomly as leaders and the rest of the population as followers. A leader makes a distancing decision as a regular player in the distancing game, while a follower just copies the strategies of some leaders unless he/she cannot find a leader in his/her group among his/her closest neighbors when he/she either makes his/her own decision or simply follows the crowd (details in **Following the leaders vs. following the crowd** in **Methods**)."

(d) The population is evenly divided into four groups. First 4 individuals are assigned to group 1,2,3,4; and then second 4 individuals are assigned to group 1,2,3,4; and so on and so forth (details in **Simulation results 2, 3** in **Supplementary Information**).
(e) There is no such a switch in the current implementation. Before each simulation, the code is manually set to take a make-own-decision strategy or a following-the-crowd strategy. It would be interesting to investigate an option for the followers to decide. It would certainly depend on more complicated decision processes.

Reviewer #1's Concern #1:

References 27-51 (lines 7-10) are collectively used within a single sentence to cover a wide range of topics related to social distancing, including the impact on mental health, economic considerations, and strategies for optimizing social activities. This does not pay sufficient tribute to the literature. A more refined discussion on the different aspects would provide a clearer and more focused analysis of the various aspects of social distancing, and thus provide a better justification of the research presented.

Response to Reviewer #1's Concern #1:

The first few paragraphs in **Introduction** are re-written, which hopefully make the points more clear. The references 27 to 51 are now separated into two groups, those from 27 to 37 on various impacts of social distancing, and from 38 to 50 on various strategies, but still not so elaborated:

"Social distancing has been advocated as an effective non-pharmaceutical measure to prevent the spread of epidemics [1-4], and has become especially well aware of to the public since the outbreak of the COVID-19 pandemic [5-13]. Social distancing plays a critical role to flat the epidemic curve before a vaccine becomes available, and remains to be so even after the population achieves certain herd immunity, for the pathogen always evolves, and the epidemic may develop into an endemic [14-22]."

"In general, social distancing or more rigorously speaking, physical distancing is referred to as for people to simply keep certain distances away from each other and avoid close in-person interactions [23-26]. However, in practice, it is not as easy to carry out as it is said. For in modern societies, social interaction is an indispensable part of human life. It is only natural for people to participate in various social or economic activities instead of avoiding them. Then, although important for fighting epidemics, social distancing comes with a price: It may cause social disruptions, economic losses, psychological stresses, etc. [27-37]"

"To carry out social distancing, one needs to manage and balance among multiple social activities – to keep some of them but reduce or completely close some others, in order to prevent the epidemic from further spreading while avoiding possible social, economic, or psychological consequences [38-50]. However, it is unclear how to optimize the activities though, often causing confusions and frustrations and resulting in overly reacted or failed distancing practices [51-57]."

The reference to Vardavas 2021 is moved to a later paragraph when the research on health behaviors in behavioral sciences is mentioned and stressed:

"Research on social distancing has surged since the outbreak of the COVID-19 pandemic, providing a wealth of knowledge and experience on non-pharmaceutical measures for preventing epidemics from spreading. However, most of these studies are about the influences of public policies, economic concerns, or cultural differences on social distancing, but not particularly about social distancing as a human behavior or the collective behavior of a population for that matter, as pointed out in a recent article by Vardavas et al. 2021 [60]. In fact, human responses to and influences on public health measures such as mask wearing, testing, and vaccination have long been investigated in behavioral sciences based on the theory of planned behavior [61,62] and the health belief models [63,64], with great insights into how health behaviors may be perceived and carried out at both individual and population levels."

Reviewer #1's Concern #2:

Line 21-22: "The decision of each individual must depend on or be influenced by the actions of all other individuals in the population. For example, if everybody decides to stay home, an individual may choose to watch a movie although the risk of having close contacts at a movie theater is high" I don't quite understand, why the decision "must" depend on or be influenced by the actions of "all other individuals". Is this only in the context of the model? A decision to watch a movie at home alone can be made just because I feel like doing it, and is thus independent of whether "all the people I know" do the same or something else. Furthermore, I don't understand the example. If everybody stays home and one person goes to the theater, the risk of getting infected is low.

Response to Reviewer #1's Concern #2:

Usually, one's decision may not necessarily depend on or be influenced by any others, but when it comes to social distancing, it is like a tragedy of commons game: if everyone is staying home, you may feel free to go shopping, because there are not so many people in grocery stores, and it is safe to go there; however, if everyone recognizes this advantage, and all come to grocery stores, which then become crowded, you would withdraw immediately from there, and go back home. This is similar to wearing a mask or not, or taking a vaccine or not, when there is a chance that you do not have to, if the majority of the population have already done it. This is also why social distancing can be modeled as a game. The word "must" may be too strong. I have changed it to "may", and also changed "go to a movie theater ..." to "go out and have dinner at a restaurant although the risk of having close contacts at a restaurant usually is high", to be more clear.

Reviewer #1's Concern #3:

I understand that for modeling purposes, things have to be simplified. However, as justification for the leader-follower scenario, I consider the statement, "Indeed, in practice, it is most likely that only a small number of individuals such as public health experts or community leaders make decisions or recommendations while all others just follow." (lines 89-91) a bold claim. I believe

that there are more factors at play and much more heterogeneity among followers of individuals.

Response to Reviewer #1's Concern #3:

Agree, the statement is too strong. I have soften it to "Indeed, in practice, it is likely that a certain number of individuals such as public health experts or community leaders make some decisions or recommendations while others follow."

Reviewer #1's Concern #4:

The same applies to "It shows how the distancing game is played out in possible realistic as well as idealistic scenarios, [. . .]" (lines 93-94). Claiming that a simulated scenario is "realistic" is a little too far. Simulations always use abstraction.

Response to Reviewer #1's Concern #4:

Agree, again. The last paragraph in **Introduction** is changed as follows:

"As such, the work in this paper confirms the game model as a plausible approach to the study of the collective behavior of social distancing. The work is still at a theoretical development stage with the model yet to be further refined against real data. It nonetheless offers some insights into how social distancing, as an adaptive social behavior, is carried out at both individual and population levels, in complex social networks, and in heterogeneous populations. It reveals the conflicting yet cooperative nature of social distancing, and sheds lights on a self-organizing, bottom-up perspective to social distancing practices."

Reviewer #1's Concern #5:

To me, the term "negative social or economic impacts" (e.g., line 123) lacks intuition and seems strange to be combined. How do these combined negative impacts come about? Something can have a positive social but negative economic impact (e.g., inviting a group of friends to an expensive restaurant).

Response to Reviewer #1's Concern #5:

It is indeed an abstract concept that tries to sum up either social or economic costs, not necessarily both. To avoid confusion, all words "social/economic" is changed to "social or economic.".

Reviewer #1's Concern #6:

It would be interesting to learn whether the times required for convergence (lines 210-214) differ depending on k. Furthermore, I would encourage the author to have a more detailed discussion of these results (i.e., what are the implications of this finding).

Response to Reviewer #1's Concern #6:

All simulation results are now for $m = 2000$, a relatively large population size. However, there is no significant difference in time when k changes. Most of the simulation converge in less than 10 generations, not depending on k or other parameters. Therefore, computation time is not recorded in the current code. For the discussion on the results, since Table 3 and Table 4 are replaced by Fig 4 and Fig 6, more detailed descriptions and discussions on the simulation results

as displayed in Fig 4, Fig 5, Fig 6 are all given in relevant sections.

Reviewer #1's Concern #7:

The statements made in lines 219-221 and especially in lines 223-225 require references for backup.

Response to Reviewer #1's Concern #7:

Wolfe 2021 and Zimmermann 2022 are cited for age differences in epidemic perception and response. Davis 2023 and Pangallo 2024 are cited for differences in economic-health tradeoff for different social groups.

Reviewer #1's Concern #8:

How were the 20 CASA activities selected? The reference to the CDC did not prove useful when I tried to reconstruct a rationale for the explicitly selected activities. Furthermore, the description of contact factors determined lacks details on methodology and data sources ("according to common practices and CDC recommendations", line 423).

Response to Reviewer #1's Concern #8:

The reference to the CDC recommendation is indeed an obscure statement. Only the safety levels of various social activities once a while announced by CDC are used to classify the CASA activities, not for determining the contact factors and impact factors. The citation is removed. The determination of contact factors and impact factors are not based on real data, but are extracted from some reasonable assumptions on how people would do for complete or free of social distancing, which hints a possible path to determining the parameters in practice. The following are some relevant paragraphs in **Results – As a population game**. More computational details are in **Deriving contact and impact factors** in **Methods**.

"Now, consider a population state, called a complete social distancing state, when every individual minimizes his/her total distancing risk over all the activities with an equilibrium strategy x^*. Given $y^* = x^*$, a corresponding set of values for w_i can be determined retrospectively (details in **Deriving contact and impact factors** in **Methods**). Set α_i to this w_i for all i. Then, a set of parameters α_i is obtained, with α_i named as the contact factor of activity i, for in a complete social distancing state, the risk of having close social contacts is presumably minimized the most by using this set of parameters."

"Then, consider another population state, called a free of social distancing state, when every individual also minimizes his/her total distancing risk over all the activities with another equilibrium strategy x^*. Given $y^* = x^*$, a corresponding set of values for w_i can be determined again retrospectively (details in **Deriving contact and impact factors** in **Methods**). Set β_i to this w_i for all i. Then, another set of parameters β_i is obtained, with β_i named as the impact factor of activity i, for in a free of social distancing state, the risk of having negative social or economic impacts from social distancing is presumably minimized the most by using this set of parameters."

"Table 1 shows the values of α_i and β_i estimated for a small set of so-called commonly attended social activities or CASA activities for short. These activities are assumed to be typical in daily small town lives in North America and are grouped into 20 general categories for the testing and simulation purposes in this work. In practice, they can certainly be extended to a much larger set of more specific and refined activities."

......

"Now, let $w_i = \delta_i \alpha_i + (1 - \delta_i)\beta_i$, $0 \leq \delta_i \leq 1$. A general set of risk factors can be obtained with δ_i called a severity parameter. If $\delta_i = 1$ for all i, $w_i = \alpha_i$. If $\delta_i = 0$ for all i, $w_i = \beta_i$. If $0 < \delta_i < 1$, w_i determines an equilibrium strategy x^* and hence y^* that corresponds to a population state between complete and free of social distancing. Based on general theory for population games, given $p_i(y) = w_i \sigma_i(y_i)$, if $w_i > 0$ for all i, an equilibrium strategy x^* for the distancing game can be obtained with $x_i^* = \sigma_i^{-1}(\lambda/w_i)$, $i = 1, \ldots, n$, assuming $x_i^* > 0$ for all i, where λ is a constant such that $\Sigma_i \, \sigma_i^{-1}(\lambda/w_i) = 1$ (details in **Equilibrium strategies and stabilities** in **Methods**)".

Reviewer #1's Concern #9:

Reference 52 calls for "spatial distancing and social closeness: not for social distancing!". Among many epidemiologists, "physical distancing" is now considered a more fitting term. I agree with the concerns raised regarding the term "social distancing" and would therefore encourage the author to reconsider the choice of terminology.

Response to Reviewer #1's Concern #9:

I would still prefer using "social distancing", for it has been used so widely in the public as well as academia, and it is understood that it is meant to keep physical (six feet) distances, not to cut social connections. To make it clear, at the beginning of **Introduction**, a line is added, saying "... In general, social distancing or more rigorously speaking, physical distancing is referred to as for people to simply keep certain distances away from each other and avoid close in-person interactions ...".

Reviewer #2's General Comments:

I am extremely sympathetic to your efforts in devising adequate policy recommendations that can help deal with future epidemics. Adjusting policies can help deal with these epidemics in a better way that what was done for the COVID-19 crisis. This can be of much value to society as a whole. However any mistake can be extremely costly and potentially fatal to individuals. So any recommendations on this topic should be strongly backed up by serious and sound analysis. While I do not doubt the seriousness of your efforts, I am not convinced by the analysis for several reasons. I believe that the simplifying assumptions (which are not discussed) available in the supplementary material, "Methods" section, are too restrictive and eliminate a number of potentially quite relevant effects.

I understand that the simulations you offer could be redone with a more precise computation of the multipliers (alpha and beta) per activity, as you mention. Providing a preliminary simulation is valuable, provided that it is made clear that these results are illustrative in nature and should not be the basis of recommendations. You do mention this before presenting the table with estimates. So this is not where the issue lays, but in the underlying model of the population game.

The core issues are with the specification of the population game and the fact that infection risk and economic benefits get added up. The remainder of the analysis, using networks and population subgroups is quite interesting and should have been a real "plus". But all the results obtained are simulated on the basis of an equilibrium which is highly unrealistic and unsatisfactory to describe an epidemic.

(The result of evolutionary stability applies only for infinitesimal perturbations to a player's strategy in the population game, and certainly not to changes in strategies in the face of shocks such as a major epidemic.)

Response to Reviewer #2's General Comments:

I really appreciate for the reviewer's patience to read the paper and for providing critical and sincere comments and suggestions! They have motivated me to re-formulate my model and helped me to revise and improve the paper substantially.

The concerns on the work, its theoretical assumptions and experimental bases, are all legitimate. It is partially due to the complex nature of social distancing, and partially due to the limit of my knowledge in social sciences as well as health sciences research. I am new to this field and will hopefully be able to learn and improve.

I am now trying to make some substantial changes in the work as well as the presentation of the paper. I have reformulated the risk assessment function $p_i(y)$ using a nonlinear logistic function. This function increases slowly at the beginning, picks up the speed after a certain threshold, and slows down again in the end, which presumably corresponds better than a linear function to the increase of the distancing risk in a given activity as the population in the activity increases. With this change, I have revised all my codes, and reproduced and documented all the computation results. The tables and graphs in **Supplementary Information** are all updated accordingly. So are the tables and graphs in the main text and the proofs in **Appendix**.

The complicated dependence of social distancing on various social, economic as well as health influences may still be oversimplified even with this change, but hopefully it will provide more flexibilities to model the social distancing behavior more accurately.

New contents are added to the paper. Many places are revised with hopefully better descriptions and interpretations. In particular, I have discussed the theories and experiments in behavioral sciences including the theory of planned behavior and the health brief models, their shared principles with the game model, and the insights that can be learned from behavioral sciences into possible improvements on the game model. I have rewritten several pages in

Results and **Methods** on the estimation of the parameters in the risk assessment functions. The estimates are not based on real data, but are extracted from reasonable assumptions on how people would spend their time for complete or free of social distancing, which hints a possible path to determining the parameters in practice. I have also taken the reviewers' suggestions and removed several numerical tables and replaced them with more accessible graphs. There are now total six figures in the paper, all regenerated except for Fig 3.

The following are my more detailed responses to Review #2's comments and concerns and the corresponding changes I have made in the paper.

Reviewer #2's Comment #1:

The simplifying assumptions in the model (cf. supplementary material) turn every distancing effect into a linear one. So the author adds linear effects, which is both quite unrealistic and mathematically problematic in the context of the study. It is highly unlikely that efforts and consequences of these efforts are linear. Moreover the linearity of all these effects lead to very specific equilibria, which are presumably quite different from the ones one would have if at least one effect was non linear. In more detail:

*A major element of the computation, function $p_i(y)$ is simply said to be (line 109, p 3) the "potential distancing risk of having close social contacts and negative social/economic impacts in activity i, when fully participated". This compounds social and economic impacts, that can be very different and potentially require different policy interventions. At the very least, this function should be discussed and explained. The function is assumed, for each activity, to be composed of two parts, a contact rate and an economic part. *But* both are assumed to be proportional only to the participating frequency y_i of the population in activity i. So everything is linear / additive. While the interpretation of the multiplier for contact rates and for social/economic aspects are different, the total effect is a sum linear in participating frequency. $p_i(y) = \delta_i \alpha_i y_i + (1-\delta_i)\beta_i y_i = w_i y_i$.*

This is highly unsatisfactory as it eliminates numerous potential effects of non linearity: It may very well be the case that in some activities, a minimal threshold of participation is necessary and / or sufficient to maintain a large part of the economic gains, or a large part of the social gains. For instance, social interactions that must be frequent enough to reach a level of friendship or a personal wellbeing. Economic activity participation is non continuous due to labor market constraints on work duration and also the need to be visible to clients and employers. This would lead to completely different strategies, where individuals could choose to be at exactly the threshold for a number of activities and at some level close to 0 for others. This appears to be a much more realistic description of the way people behaved during the COVID-19 crisis. This is important to understand as it means that more efforts should be put in providing adequate protection from the epidemics in some activities that both have threshold values to obtain benefits and have high benefits to individuals. For instance invest in distancing widgets or remote technologies to provide these benefits for these activities, and not invest in others where such discontinuous effects do not exist.

Response to Reviewer #2's Comment #1:

Thanks for pointing this out. The dependence of distancing risk on the population size in a given activity is indeed oversimplified by using a linear function. I have tried several nonlinear functions and ended up using a logistic function to define this dependence. This function increases slowly at the beginning, picks up the speed after a certain threshold, and slows down again in the end, which presumably corresponds better than a linear function to the increase of the distancing risk in a given activity as the population in the activity increases. The following are the paragraphs added in the section on **As a population game** in **Results** for the description of this function,

with new contents all colored in blue.

"Assume that the social activities are independent of each other, i.e., the individuals participating in one activity do not have contacts with those in other activities. Then, the function $p_i(y)$ can be defined to depend only on the participating frequency y_i of the population in activity i. Let $p_i(y) = w_i \sigma_i(y_i)$, where w_i is a constant called the risk factor of activity i, and σ_i is a logistic function of y_i, typically increasing slowly when y_i is in a low range, picking up the speed after y_i passes a certain threshold, and slowing down again when y_i enters in a high range (as shown in Fig 1), which presumably corresponds to how the distancing risk at a given activity increases with increasing participating frequency of the population in that activity."

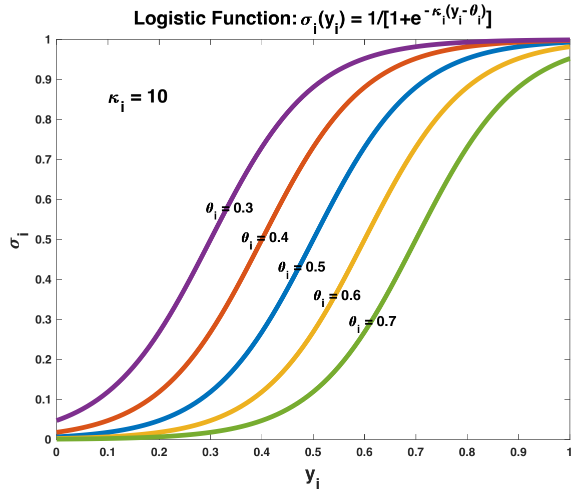

Fig 1. Logistic functions $\sigma_i(y_i)$ with a rate constant $\kappa_i = 10$ shifted by $\theta_i = 0.3, 0.4, 0.5, 0.6, 0.7$, all increasing slowly when y_i is in a low range, picking up the speed after y_i passes a certain threshold, and slowing down again when y_i enters in a high range.

The logistic function has two parameters, κ_i and θ_i. The parameter κ_i determines the increasing rate of the function. If it is increased, the beginning and ending parts of the function would become flatter. The parameter θ_i makes a shift to the function, and can be used to adjust the increasing threshold of the function. For different activities, the risk assessment function may have different κ_i and θ_i values to fit the increase of their distancing risks. However, in this paper, I have only considered a general set of activities. It is hard to decide the κ_i and θ_i values for them specifically. Therefore, I have simply set $\kappa_i = 10$ and $\theta_i = 0.5$ for all the activities for easy to analyze and simulate.

The contact and impact factors α_i and β_i in the risk assessment function are not determined with real data, but are extracted from some assumptions on how the population would spend time on each of the activities in either a complete social distancing state or a free of social distancing state. The following are the descriptions modified in the section on **As a population game** in **Results** on how the two sets of parameters are determined and what they imply in the assessment of the distancing risks.

"Now, consider a population state, called a complete social distancing state, when every individual minimizes his/her total distancing risk over all the activities with an equilibrium strategy x^*. Given $y^* = x^*$, a corresponding set of values for w_i can be determined retrospectively (details in **Deriving contact and impact factors** in **Methods**). Set α_i to this w_i for all i. Then, a set of parameters α_i is obtained, with α_i named as the contact factor of activity i, for in a complete social distancing state, the risk of having close social contacts is presumably

minimized the most by using this set of parameters."

"Then, consider another population state, called a free of social distancing state, when every individual also minimizes his/her total distancing risk over all the activities with another equilibrium strategy x^*. Given $y^* = x^*$, a corresponding set of values for w_i can be determined again retrospectively (details in **Deriving contact and impact factors** in **Methods**). Set β_i to this w_i for all i. Then, another set of parameters β_i is obtained, with β_i named as the impact factor of activity i, for in a free of social distancing state, the risk of having negative social or economic impacts from social distancing is presumably minimized the most by using this set of parameters."

"Table 1 shows the values of α_i and β_i estimated for a small set of so-called commonly attended social activities or CASA activities for short. These activities are assumed to be typical in daily small town lives in North America and are grouped into 20 general categories for the testing and simulation purposes in this work. In practice, they can certainly be extended to a much larger set of more specific and refined activities."

......

"The first eight CASA activities include 1. reading/watching TV, 2. work at home, 3. hiking, 4. gardening, 5. stay with family, 6. grocery shopping, 7. go to hospitals, 8. visit friends. They have low contact rates and hence relatively low contact factors. The last eight CASA activities include 13. watch sports, 14. attend concerts, 15. go to schools, 16. go to workplaces, 17. large gathering, 18. go to bars or night clubs, 19. air traveling, 20. go to movie theaters. They have high contact rates and hence relatively high contact factors. In between, there are also four frequently participated CASA activities, 9. restaurant/cafeteria dinning, 10. go to shopping malls, 11. take buses, 12. go to churches. Their values of contact factors are at a moderate level."

"The values for the impact factors are not necessarily negatively correlated with the values of the contact factors. They vary in a small range except for activities 15. go to schools and 16. go to workplaces whose impact factors are quite small. The smaller the impact factor, the less negative social or economic impact in the activity or in other words, the more socially or economically favorable. For example, although in the same level of contact factors, activities 3. hiking and 4. gardening have relatively larger impact factors than activities 1. reading/watching TV and 2. work at home which seem to be more socially or economically favorable. On the other hand, although activities 17. large gathering and 18. go to bars or night clubs have relatively large contact factors, their impact factors are smaller than most of the activities because of their high social demands. The impact factors for activities 15. go to schools and 16. go to workplaces are among the smallest, agreeing with the fact that they are certainly two of the most important social and economic activities in modern human lives."

......

"Now, let $w_i = \delta_i \alpha_i + (1-\delta_i)\beta_i$, $0 \le \delta_i \le 1$. A general set of risk factors can be obtained with δ_i called a severity parameter. If $\delta_i = 1$ for all i, $w_i = \alpha_i$. If $\delta_i = 0$ for all i, $w_i = \beta_i$. If $0 < \delta_i < 1$, w_i determines an equilibrium strategy x^* and hence y^* that corresponds to a population state between complete and free of social distancing. Based on general theory for population games, given $p_i(y) = w_i \sigma_i(y_i)$, if $w_i > 0$ for all i, an equilibrium strategy x^* for the distancing game can be obtained with $x_i^* = \sigma_i^{-1}(\lambda/w_i)$, $i = 1, \ldots, n$, assuming $x_i^* > 0$ for all i, where λ is a constant such that $\Sigma_i \, \sigma_i^{-1}(\lambda/w_i) = 1$ (details in **Equilibrium strategies and stability** in **Methods**)."

Note that with the risk assessment function defined in terms of the logistic function, the equilibrium strategy can be computed in terms of the inverse logistic function. I have checked and compared the equilibrium strategies computed in this way with those previously obtained by

using the linear function. The participating frequencies for "high risk" activities such as 15. go to schools and 16. go to workplaces in the new equilibrium strategies become significantly higher than previously calculated. The new risk assessment seems indeed tolerate more population increases in these activities as long as they do not exceed certain threshold values. This is an important property for the risk assessment function to possess, as indicated by the reviewer.

Reviewer #2's Comment #2:

An obvious additional non-linearity is the one that arises from budget / subsistence constraints : Many people need to work in order to meet their subsistence requirements. The strength of this constraint and the number of people strongly affected by it depend crucially on regional characteristics pre-epidemic but also on policy responses to the epidemic.

Response to Reviewer #2's Comment #2:

Yes, agree. There could be various types of constraints on the distancing strategies. In terms of the time spent on the activities, there may be lower and upper bounds on the time for some of them. For example, for essential workers, they may have to go to workplaces for at least a small amount of time; and a person cannot stay in a restaurant for more than two hours in one visit. On the other hand, some activities may need to be fixed to zero. For example, seniors do not go to bars; and children do not go to work. The economic constraints can be made even more specific or subtle. However, all such constraints have not be included in the current work. They certainly need to be considered in future efforts. With that I have added a new paragraph in **Discussion** to bring this issue up:

"When choosing among a group of activities, there must be personal constraints for individuals to attend some of them. For example, some people may have to go to hospital at least once a week; the essential workers or kindergarten teachers may have to go to work for a minimum amount of time. There must be certain time limits for some activities as well such as air traveling or even restaurant dinning. On the other hand, some individuals may never attend some of the activities. For example, children may never go to workplaces while seniors would hardly go to bars or schools. Such constraints have not been implemented in the current model in the paper. They should be included in the further expansion of the investigation in future."

Reviewer #2's Comment #3:

More generally in many situations gains are concave (diminishing returns). This also leads to different equilibria and potentially multiple equilibria, a situation in which coordination issues would be of primary interest. The role of networks and leaders would take a much different shape then than in the analysis provided here.

Response to Reviewer #2's Comment #3:

Agree, again. These are important issues yet to be addressed, but in this work, in some sense, they are purposely avoided to focus on some other issues with the distancing game. They cannot be avoided in practice, however, if a complete and realistic model is to be built eventually. The following paragraph is added in **Discussion** as a comment on these issues.

"The distancing game must have multiple equilibrium strategies in general, which have not been addressed in this work. This could happen when more realistic risk assessment functions are used, for example, when a different δ_i is set to a different activity or a different logistic function σ_i is used for a different activity with different κ_i and θ_i values. It could happen when constraints are introduced on the participating frequencies in some of the activities. It could also

happen when some of the activities are dependent of each other as shown in previous studies [90,91]. Social distancing is a complex social behavior and cannot be modeled successfully until all these factors are considered."

Reviewer #2's Comment #4:

The values are computed in a way that amounts to adding up "apples and oranges" : p_i is the sum of risks from being close to other, potentially infectious, individuals, and of economic and social costs to avoiding activities. It makes no sense to add them, and could only correspond to a highly specific form of the individuals' utility function, eliminating for instance risk aversion, decreasing returns, complementarities between health status and economic and social benefits, etc. It cannot in any way represent risks that can vary with individual efforts, as these efforts have a different impact on the infection risk than on the economic impact. One cannot study an epidemic, which has a dynamic nature and is the source of specific anxiety to individuals, with parameters that sum infection risk and economic aspects.

In particular, the cost associated to a higher contact rate (the "alpha" part in the model) depends highly on the disease frequency in the population. The propagation of the epidemic follows a specific dynamic, endogeneous to individual activity choices, and the infection risk associated to a contact rate is not constant over time.

These assumptions are stated but not discussed in the paper, but they are crucial for the results. The topic is of much importance given epidemiologists' concerns about future epidemics. I hope that some improvement can be done by building a different model. However the dynamics of the epidemic and the endogeneity of these dynamics to individual choices make this a highly difficult task. I unfortunately cannot suggest easy changes that would answer my concerns, but I do hope that you will find a way to build a different analysis with the same objective of analyzing the impact of networks. I think it would be basically a different paper. If only one aspect is treated, at the very least the risks associated to a contact rate cannot be linear, cannot have a constant marginal value, in the face of an evolving epidemic, and cannot be added to economic benefits in a linear way. I hope that you will continue your efforts (that have been successful in the past) but with a better modeling of individual choices as a standard population game may not be suited here.

Response to Reviewer #2's Comment #4:

As in the **Response to Reviewer #2's Comment #1**, I have tried to provide a better explanation on the combination of the contact and impact risks in the risk assessment function. It may not be a simple addition of close social contacts and social or economic impacts. It is a convex combination of the distancing risks in two extremum population states: One is the so-called complete social distancing state when the total amount of close social contacts is presumably minimized the most. Another is the so-called free of social distancing state when the total amount of negative impacts is presumably minimized the most. The combination of the two components gives an assessment on the distancing risks for a population state in between the two extreme states. The contact and impact factors α_i and β_i are not determined with real data, but are extracted from some assumptions on how the population would spend time on each of the activities in either a complete social distancing state or a free of social distancing state. The following are the descriptions modified in the section on **As a population game** in **Results**.

"Now, consider a population state, called a complete social distancing state, when every individual minimizes his/her total distancing risk over all the activities with an equilibrium strategy x^*. Given $y^* = x^*$, a corresponding set of values for w_i can be determined retrospectively (details in **Deriving contact and impact factors** in **Methods**). Set α_i to this w_i for all i. Then, a set of parameters α_i is obtained, with α_i named as the contact factor of activity i,

for in a complete social distancing state, the risk of having close social contacts is presumably minimized the most by using this set of parameters."

"Then, consider another population state, called a free of social distancing state, when every individual also minimizes his/her total distancing risk over all the activities with another equilibrium strategy x^*. Given $y^* = x^*$, a corresponding set of values for w_i can be determined again retrospectively (details in **Deriving contact and impact factors** in **Methods**). Set β_i to this w_i for all i. Then, another set of parameters β_i is obtained, with β_i named as the impact factor of activity i, for in a free of social distancing state, the risk of having negative social or economic impacts from social distancing is presumably minimized the most by using this set of parameters."

"Table 1 shows the values of α_i and β_i estimated for a small set of so-called commonly attended social activities or CASA activities for short. These activities are assumed to be typical in daily small town lives in North America and are grouped into 20 general categories for the testing and simulation purposes in this work. In practice, they can certainly be extended to a much larger set of more specific and refined activities."

An important issue brought up in this comment is the interdependence between the dynamic changes of the epidemic and the changes of social distancing behaviors while "the infection risk associated to a contact rate is not constant over time". There is no estimate on the constant dynamic changes of the infection rate and how they affect the social distancing behaviors in this work. However, there are well developed epidemiological models for predicting the dynamic changes of the infection rate in an epidemic, given a presumed contact rate. Therefore, it is possible to combine the model for social distancing with an epidemiological model to obtain a more complete description on how social distancing behaviors are correlated with the epidemic developments dynamically. The following is a new paragraph added in **Discussion** on this subject.

"Finally, the social distancing behavior depends on the severity of the epidemic, while the latter is not constant and also depends on the former. For example, when the severity of infection is high, social distancing must be enhanced; when the severity of infection is low, social distancing may be relaxed. Such changes may happen back and forth in the entire epidemic time. They may in turn influence the dynamics of epidemics as well. Therefore, a complete model for social distancing must be coupled with an epidemiological model so that the changes of social distancing behaviors can be predicted successfully with changing epidemiological conditions and vice versa, as called for research on incorporating human behavior in epidemiological models in a recent US NSF program announcement [118]."
